# Mechanistic insights into nitrogen fertilizer regulation of carbon-nitrogen cycling and greenhouse gas emissions: a metagenomics-based investigation

**DOI:** 10.3389/fmicb.2026.1808047

**Published:** 2026-04-17

**Authors:** Tiantian Meng, Jingjing Shi, Xiangqian Zhang, Xiaoyu Zhao, Yanan Liu, Meiren Rong, Liyu Chen, Yu Dai, Shuli Wei, Jiawei Liu, Zhanyuan Lu

**Affiliations:** 1 College of Agronomy, Hebei Agricultural University, Baoding, China; 2 Inner Mongolia Academy of Agricultural and Animal Husbandry Sciences, Hohhot, China; 3 School of Life Science, Inner Mongolia University, Hohhot, China; 4 Inner Mongolia Key Laboratory of Degradation Farmland Ecological Restoration and Pollution Control, Hohhot, China; 5 Key Laboratory of Black Soil Protection and Utilization (Hohhot), Ministry of Agriculture and Rural Affairs, Hohhot, China

**Keywords:** carbon–nitrogen cycle, greenhouse gasses, metagenomics, microbial diversity, nitrogen fertilizer

## Abstract

Nitrogen (N) fertilizer application can regulate the structure of soil microbial community and influence the abundance of functional genes involved in carbon (C) and N cycling, thereby affecting greenhouse gas (GHG) emissions. This study was conducted in 2023–2024, setting up six nitrogen application rates: N0 (0 kg·ha^−1^), N120 (0 kg·ha^−1^), N180 (0 kg·ha^−1^), N240 (0 kg·ha^−1^), N300 (0 kg·ha^−1^), and N360 (0 kg·ha^−1^). Using 16S amplicon sequencing technology and metagenomic sequencing, the study analyzed the abundance of carbon and nitrogen cycling functional genes. Combined with measurements of CH₄, N₂O, and CO₂ emission fluxes, the research elucidated the mechanism by which nitrogen fertilizer regulates microbial modulation of greenhouse gas emissions. The results indicated that nitrogen application significantly increased greenhouse gas (CH₄, N₂O, CO₂) emissions, with the highest emissions observed under the N300 treatment. Nitrogen application regulated soil nutrients, increasing soil total nitrogen, nitrate nitrogen, and microbial biomass carbon content. Reasonable nitrogen application (N240) increased bacterial *α*-diversity (Shannon index, Chao index, PD index) in the soil by 10.82, 14.65, and 1.92%, respectively, compared to N0. It also increased the abundance of dominant nitrogen-fixing bacterial phyla, including Actinobacteria, Proteobacteria, and Nitrospirota. Furthermore, it regulated the abundance of microbial-mediated functional genes involved in dissimilatory nitrate reduction (*nirB*), assimilatory nitrate reduction (*nasA*), denitrification (*narG*, *narH*, *nirS*), nitrification (*norC*, *nxrA*, *nxrB*, *hao*, *amoC*), as well as those in the carbon cycle related to methane metabolism (*pmoA*, *pmoC*, *mttC*), carbon fixation (*por/nifj*, *rbcl/cbbl*), and hydrogenotrophic methanogenesis (*mch*, *hdrA*, *frdE*). This regulation further modulated greenhouse gas emissions. Therefore, this study clarifies the microbe-associated mechanisms underlying the N fertilizer-driven coupling of C and N cycles with GHG emissions through an integrated analysis of microbial diversity and metagenomics. Furthermore, it offers new insights for sustainable N fertilizer management and emission mitigation strategies in agricultural systems.

## 1 Introduction

Farmland is an important ecosystem that supports food security and human health and is one of the largest nitrogen (N) flow carriers on Earth. The continuous acceleration in climate change and greenhouse gas (GHG) emissions has exacerbated the vulnerability of farmland systems, thereby threatening global food and ecological security (Cui et al., 2023). GHG emissions from agricultural ecosystems account for 23% of the total terrestrial ecosystem emissions (Zhao et al., 2021). Carbon dioxide (CO_2_) accounts for 66% of the total radiation intensity of GHGs, with prolonged survival, and has become the largest contributor to global warming (Friedlingstein et al., 2023). Methane (CH_4_) is the second most crucial GHG after CO_2_, with a century-scale warming potential of 27.9 times that of CO_2_ (Intergovernmental Panel on Climate Change, 2021), which can hugely impact global climate change (Han and Chen, 2020). Nitrous oxide (N_2_O) is the third most abundant GHG and has the longest residual time in the atmosphere. Its warming potential measured over the past century is 273 times that of CO_2_ (Intergovernmental Panel on Climate Change, 2007). It is well-established that the application of N fertilizer influences crop growth and carbon gas (CO₂ and CH₄) emissions (National Coordination Committee on Climate Change, 2012; Guan et al., 2019). However, its role in stimulating the emission of N₂O represents a major environmental tradeoff (Qiu et al., 2020). The global increase in N₂O, largely arising from crop production and N fertilizers (Azeem et al., 2014), is mechanistically linked to the stimulation of microbial N transformation, most notably nitrification and denitrification, in agricultural soils (Zhu et al., 2014; Zou et al., 2009).

Soil microorganisms are huge in quantity and composite in community structure, with complex interactions among various microorganisms, directly participating in all links between the C and N cycle-related transformations (Kuzyakov and Blagodatskaya, 2015). The application of N fertilizers affects the C and N cycles through soil microorganisms, thereby changing the original GHG emission levels (Feng X. et al., 2025; Feng Y. et al., 2025). Soil microorganisms actively participate in the decomposition and transformation of organic matter through various metabolic pathways, playing a crucial role in the C cycle within the soil system. They contribute to organic C stabilization, thereby influencing soil C storage and turnover (Wu et al., 2024). N fertilizer application regulates microbial respiratory substrates and CO₂ emissions by influencing the functional genes associated with organic matter decomposition and carbon fixation, such as *cbbL*, which encodes the large subunit of ribulose-1,5-bisphosphate carboxylase/oxygenase (RuBisCO) (Zhou et al., 2018). N fertilizers can increase the quantity of C substrates and promote the growth of soil functional microorganisms, such as methanogens and methanotrophs (Banger et al., 2012). Meanwhile, O_2_ combines with NH₄^+^-N generated from N fertilizer mineralization, further promoting the oxidation of CH₄ (Qian et al., 2022); most of is converted into CO₂ and H₂O by methanotrophs (Islam et al., 2019). Microorganisms decompose total organic C to produce CO₂, which can also be reduced to CH₄ by methanogens (Borrel et al., 2017). Soil properties can affect GHG emissions by stimulating the proliferation of bacteria involved in CH₄ production, nitrification, and denitrification (Yao et al., 2023). The remarkable enrichment of *pmoA*, *pmoC*, and *mttC* during CH₄ metabolism affects CH₄ emissions, and N fertilizers reduce the abundance of methanogenic (*mttB* and *mttC*) and CH_4_-oxidizing (*pmoA* and *pmoB*) genes (Zhang et al., 2023a,b,c; Zhang X. et al., 2023). Methanogenesis (*mch*, *hdrA*, and *frdE*) produces CH₄ from simple compounds, such as CO_2_, methanol, and acetic acid (Wang et al., 2022). N fertilizers can regulate the abundance of functional genes involved in C and N cycles by influencing the soil C and N nutrient contents.

The application rate of N fertilizer induces a direct regulatory influence on the abundance and activity of key functional genes that are involved in these processes (Kuypers et al., 2018). Biological nitrogen fixation, mediated via the nitrogenase complex, is primarily governed by the gene abundance of structural genes such as *nifH* and *nifD*, which directly determine the ecosystem N input and provide substrates for subsequent N transformation (Jiang et al., 2021). However, N fertilization has been demonstrated to directly suppress the abundance of these nitrogen-fixation genes, thereby reducing the biological nitrogen fixation (Dijkstra et al., 2010). Ammonium (NH₄^+^), which is introduced into the soil, is predominantly oxidized via nitrification. This process is initiated by *amoA*, *amoB*, and *amoC* encoding ammonia monooxygenase, followed by the oxidation of hydroxylamine (NH₂OH) to nitrite (NO₂^−^), which is catalyzed by the enzyme encoded by *hao*. This pathway represents a substantial source of N₂O emission in agricultural soil (Wright et al., 2020; Wu et al., 2021; Zhang et al., 2023a,b,c; Zhang X. et al., 2023). Numerous studies have demonstrated that low-to-moderate levels of N fertilizer application substantially enhance the abundance of *amoA* and *hao*, thereby stimulating nitrification and subsequently increasing N₂O emission (Hu et al., 2013; Li D. et al., 2015; Li J. et al., 2015). Nitrification is a crucial process leading both directly and indirectly to N_2_O production. N fertilizer application elevates soil nitrate (NO₃^−^) concentration, which in turn stimulates the abundant abundance of *narG* and *nirS*/*nirK*; thus, it is a major contributor to the increase in GHG emissions (Zhou X. et al., 2024; Zhou Z. et al., 2024). In contrast, assimilatory nitrate reduction (ANRA), mediated by genes such as *nasA* and *nirA* (Lledó et al., 2005), and dissimilatory nitrate reduction to ammonium (DNRA), catalyzed by enzymes encoded by *napA* and *nrfA* (Mauffrey et al., 2015), represent competing nitrate utilization pathways. Research indicates that N fertilizer application can influence the DNRA process; an enhanced DNRA activity can reduce the nitrate pool available for denitrification, thereby potentially mitigating N₂O emissions indirectly (Friedl et al., 2018).

As a typical ecological transition zone, the agro-pastoral ecotone of Inner Mongolia serves as a critical base for grain and livestock production in northern China. Consequently, this region faces substantial pressure to mitigate greenhouse gas emissions while safeguarding national food security (Gao et al., 2019). However, research remains limited on how nitrogen application modulates soil microbial communities and their associated carbon and nitrogen cycling functions to influence greenhouse gas emissions. Most studies have mainly focused on the impacts of different N fertilizer levels on bacterial community structure and associated functional genes. However, research on the mechanisms underlying the relationships among GHG emissions, N fertilizers, soil, and microbial ecological functions is still lacking. This study integrated soil microbial diversity with metagenomics under varying N fertilizer application levels to assess the relative abundance of genes involved in C and N cycling and their feedback relationships with *in situ* GHG emission fluxes. Using metagenomic component box technology, we identified GHG emission–associated functional genes within bacterial communities and elucidated the impacts of N fertilizers on soil microecology and GHG emissions. Therefore, this study hypothesizes that: (i) Through comparative analysis of soil physicochemical properties and microbial diversity under different N fertilizer application treatments, the assembly patterns and differential bacterial communities of the soil bacterial community will be elucidated; (ii) By integrating soil metagenomics with greenhouse gas emission data, the key metabolic pathways and functional genes regulating GHG emissions will be identified, and the core bacterial communities associated with C and N cycling will be determined; and (iii) Multifactor integrated analysis will clarify the response patterns of microbial communities to varying N fertilizer application rates, thereby revealing the mechanisms by which microorganisms mediate the effects of N fertilizer on GHG emissions in maize fields.

## 2 Materials and methods

### 2.1 Test site

**Figure 1:**
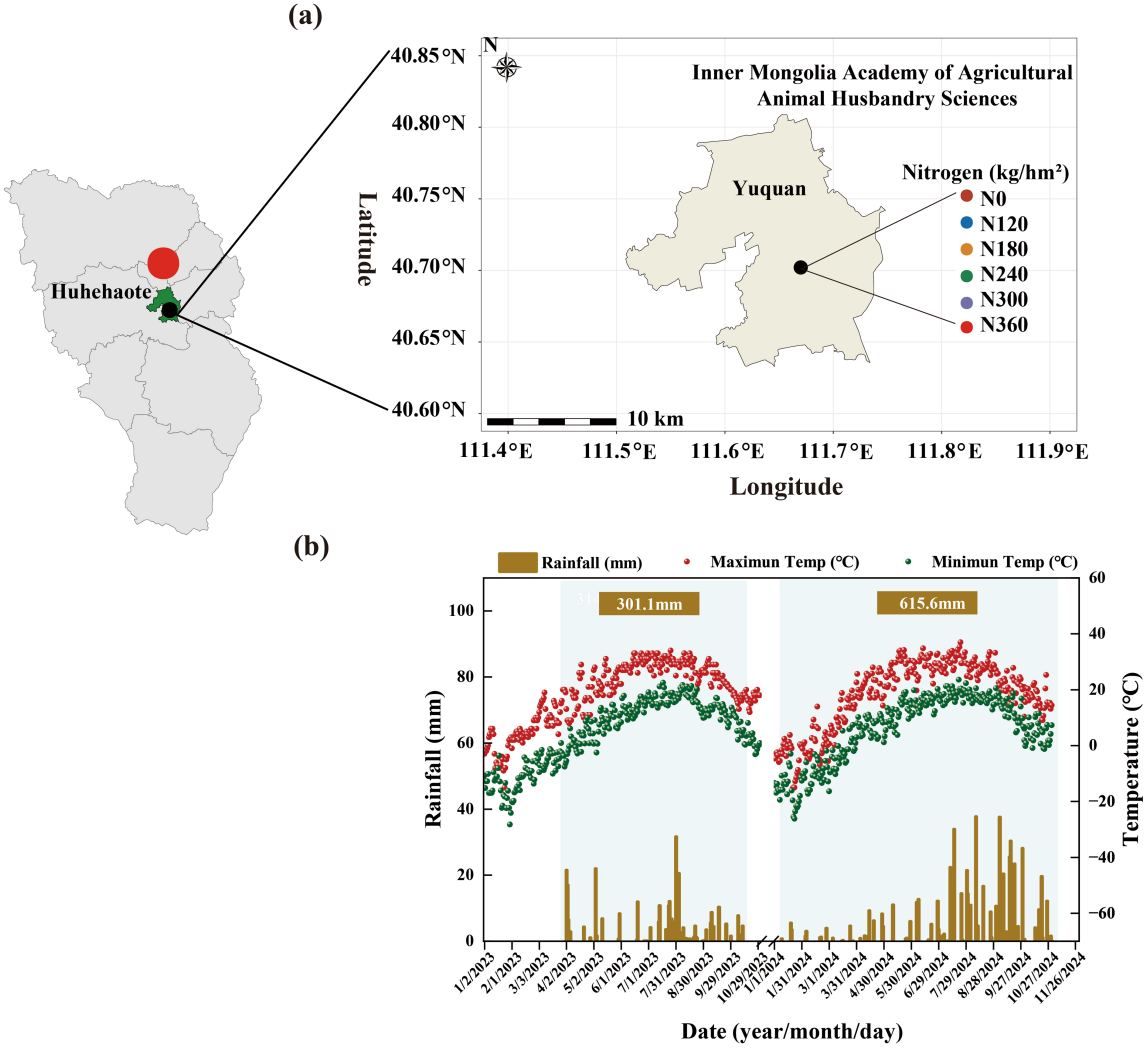
Overview of the experimental site. **(a)** Location of the experimental site; **(b)** precipitation, maximum temperature, and minimum temperature at the experimental site from January to October in 2023–2024. The blue shaded area in the figure represents the entire growth period of maize.

This study was conducted from 2023 to 2024 at the experimental base of the Inner Mongolia Academy of Agricultural and Animal Husbandry Sciences, Hohhot, China (Figure 1). The experimental site is within an agropastoral ecotone. During the entire maize growth period, rainfall was mainly concentrated from July to September (Figure 1). The annual evaporation was 1,531 mm; the annual sunshine was 2,764 h; the frost-free period ranged from 113 to 134 days; and the average temperature from May to October was 17.2 °C. The soil type was loamy, and the previous crop cultivated was maize. Before the start of the experiment in 2018, the basic physiocochemical and nutrient status of a 0–20 cm layer of the soil was determined as follows: pH: 7.62, total nitrogen (TN): 1.08 g·kg^−1^, total phosphorus (P): 0.77 g·kg^−1^, total potassium (K): 0.36 g·kg^−1^, available P: 15.92 mg·kg^−1^, available K: 117.50 mg·kg^−1^, alkaline hydrolyzable N: 59.50 mg·kg^−1^, and organic matter: 22.63 g·kg^−1^.

### 2.2 Experimental design

This study was initiated in 2018 and conducted from 2023 to 2024. It involved a long-term field fertilization experiment and *in situ* observations to analyze the effects of different N fertilizer application rates on microorganism-mediated GHG emissions and C–N cycling processes. A single-factor randomized block design was adopted. The maize variety ‘Guangde 5’ was selected as the test material. In total, six N fertilizer application levels were set: N0 (0 kg·ha^−1^), N120 (120 kg·ha^−1^), N180 (180 kg·ha^−1^), N240 (240 kg·ha^−1^), N300 (300 kg·ha^−1^), and N360 (360 kg·ha^−1^). Each treatment was replicated thrice, in 18 plots, each covering an area of 27.9 m^2^. The equidistant row-planting method was employed, with a row spacing of 0.6 m, plant spacing of 22.2 cm, and protective two rows, each 1 m wide.

Manual sowing was employed, and the sowing density was 75,000 plants·ha^−1^. Before sowing, the same amounts of P (300 kg·ha^−1^ P₂O₅) and K (120 kg·ha^−1^ K₂SO₄) fertilizers were applied for each treatment. Resin-coated urea with 45% N content was used as the N fertilizer. Manual weeding and pest control were performed during the growth stage. Drip irrigation was performed using a main drip pipe with a 60 mm diameter. The water volume was recorded with a water meter, which was 425.00 m^3^·ha^−1^ in June, July, and August. The other field management practices were the same as those applied in large-scale fields.

### 2.3 Sample collection and analysis

#### 2.3.1 Soil sample collection

Soil sampling sites in the maize fields were selected according to the soil GHG emission characteristics. Surface soil samples (0–10 cm depth) were collected on June 28, 2023, and June 29, 2024 (Prajuli et al., 2025). The collection method employed was as follows: within each plot, five soil cores along the “S”-shaped curve were collected, mixed, and used as a representative sample. A 2-mm sieve was used to screen the soil samples and remove impurities such as roots and stones. Then, the samples were divided into two parts; one part was air-dried and brought back to the laboratory in a self-sealing bag. This sample was ground and sieved in a cool place. The filtered powder is used for physicochemical analyses. Another part was stored at −20 °C for determining soil nitrate N (NO_3_^−^-N), ammonium N (NH_4_^+^-N), and microbial biomass C and N. Soil samples between the maize rows were collected and sieved to 1 mm–sized particles. After flash-freezing in liquid N_2_, the filtered samples were used for microbial diversity and metagenome analysis. Sequencing was performed with three biological replicates, and the metagenomic sequencing depth was 12 Gb per sample.

#### 2.3.2 Determination of soil physical and chemical properties

The bulk density method was employed to determine the water content. The temperature was measured using a TRIME-PICO64/32 TDR portable soil moisture meter (Auzuo Ecology Instrumentation Ltd., Beijing, China). The inorganic nitrogen content was determined through extraction with 1 M KCl and examined with an AA3 HR Continuous Flow Autoanalyzer (Seal Analytical GmbH, Norderstedt, Germany) (Zheng et al., 2013). The pH was measured using a CyberScan pH 510 pH meter (Thermo Fisher Scientific, MA, United States) at a water: soil ratio of 5:1. TN was assessed via digestion with concentrated H₂SO₄ and using a Kjeltec TM 8400 Kjeldahl N analyzer (FOSS, Hillerød, Denmark) (Sparks et al., 1996). Soil organic carbon (SOC) was measured using the potassium dichromate oxidation method. Samples were titrated against a standard FeSO₄ solution using an IS Digital electronic titrator (BRAND GMBH+CO KG, Wertheim, Germany). Soil microbial biomass C and N were determined using the chloroform fumigation–extraction method (Zhang et al., 2023a,b,c; Zhang X. et al., 2023).

#### 2.3.3 Determination of N₂O, CO₂, and CH₄

The static chamber method was used to determine the emission fluxes of GHGs (CO₂, CH₄, and N₂O) in maize plants from sowing to harvest under different agricultural management practices. The gas collection chamber (30 × 30 × 60 cm) was made of an acrylic material. After sowing, three static chamber bases were installed in each treated field for triplicate measurements. The bases were buried between the crop rows, 5 cm into the soil, and remained stationary throughout the growing season. During sampling, the plants were removed from the grooves of the bases; the sampling chambers were placed vertically in the grooves and sealed with water to ensure no atmosphere–chamber gaseous exchange. Before sampling, a syringe was inserted into the chamber and pumped 5–10 times to uniformly mix the gases. At 10, 20, and 30 min after chamber closure, the gas samples were extracted from the chamber with a 50-ml syringe and injected into vacuum tubes for subsequent analysis.

The gas samples were analyzed using a 7890A gas chromatograph (Agilent Technologies, Inc., CA, United States) with automatic injection to detect the integral areas of CO₂, N₂O, and CH₄. The concentrations of the target gases in the samples were estimated based on the known concentrations and response areas of standard CO₂, N₂O, and CH₄ gases provided by the National Institute of Metrology, Beijing, China. CH₄ was separated on a Porapak Q packed column and identified using a flame ionization detector (FID). CO₂ was split on a Porapak Q packed column, reduced using a nickel converter, and then detected using FID. N₂O was identified using an electron capture detector (μECD). The operating temperatures of the nickel converter, FID, and μECD were 375 °C, 200 °C, and 330 °C, respectively, whereas the oven temperature was 55 °C. Gas was sampled from 09:00 to 11:00 a.m. on June 28, 2023, and June 29, 2024 (Peak emission flux stage). Greenhouse gas emission fluxes were calculated based on the mean values derived from multiple sampling events conducted over 2 years.

N_2_O, CO_2_, and CH_4_ emission fluxes were calculated using Equation 1 (Wang J. et al., 2024; Wang X. et al., 2024):


$$F=\rho \times h\times \frac{\mathrm{dc}}{\mathrm{dt}}\times \frac{273}{273+T}$$ (1)

Where 𝐹 is the GHG emission flux in mg m^−2^ h^−1^; *ρ* is the gas density in its standard state; h is the height of the static box (m); $\frac{\mathrm{dc}}{\mathrm{dt}}$ is the rate of change of GHG concentrations in the static box (a linear fit was used, with *R*^2^ detecting the goodness of fit), and T is the temperature of the gas chamber during the sampling period (°C).

#### 2.3.4 Determination of soil bacterial community diversity and composition

The genomic DNA of the total microbial community was extracted using the E. Z. N. A.^®^ soil DNA kit (Omega Bio-tek, GA, United States) according to the instructions. DNA concentration and purity were determined, and integrity was verified via 1% agarose gel electrophoresis. The DNA was fragmented using Covaris M220 (Majorbio, Co., Ltd., Shanghai, China), and fragments, approximately 350-bp long, were selected for constructing the paired-end library. The library was prepared using the NEXTFLEX Rapid DNA-Seq kit (Bioo Scientific Corporation, TX, United States). The soil amplification primer of the bacterial 16S gene was 343F: 5′-TACGGRAGGCAGCAG-3′ and 806R: 5′-AGGGTATCTAATCCT-3′ (Xu et al., 2016). It involved adapter ligation, removal of the self-ligated adapter fragments via magnetic bead selection, enrichment of library templates through PCR, and recovery of the amplicons employing magnetic beads to obtain the final library. Metagenomic sequencing was performed on the NovaSeq™ X Plus platform (Illumina) at the Shanghai Majorbio Bio-pharm Technology Co., Ltd., Shanghai, China (Wei et al., 2023).

The V3–V4 hypervariable region of the bacterial 16S rRNA gene provides comprehensive coverage and maximum taxonomic accuracy for classification based on bacterial genome sequence. PCR amplification of the V3–V4 fragment was performed using specific primers. The amplicons were recovered using 2% agarose gel electrophoresis, purified with an AxyPrep DNA Gel Extraction Kit (Axygen Biosciences, CA, United States), detected via 2% agarose gel electrophoresis, and quantified using a Quantus™ Fluorometer (Promega, WI, United States). Libraries were constructed with the NEXTFLEX Rapid DNA-Seq Kit (Revvity Health Sciences, Inc., MA, United States) and sequenced on an MiSeq PE300 platform (Illumina, Inc., CA, United States). After quality control, splicing, and chimera removal of the raw sequencing reads, operational taxonomic units (ASV) were clustered at 100% similarity using USEARCH Version 7.1^1^. Species annotation based on each 16S rRNA gene sequence was performed using the RDP classifier algorithm with a 70% confidence threshold (Fang et al., 2025). The raw sequences were deposited in the SRA database (Accession Nos. PRJNA1291659 and PRJNA1293101).

### 2.4 Metagenomic determination

#### 2.4.1 Sequence quality control, genome assembly, and functional annotation

Data quality control was performed by trimming the adapter sequences at the 3′ and 5′ ends of the reads using fastp Version 0.20.0^2^ (Chen et al., 2018). After trimming, reads <50 bp and those with an average base quality score <20 were removed, thereby retaining the high-quality sequences. The reads were aligned to the host DNA sequences using BWA Version 0.7.17^3^ (Li and Durbin, 2009), and contaminations with high similarity were filtered (if the samples were derived from a host, such as human or animal feces, and the genome sequence was published).

The optimized sequences were assembled using MEGAHIT Version 1.1.2^4^ (Li D. et al., 2015; Li J. et al., 2015). Contigs ≥300 bp were selected from the assembled sequences as the final output. ORFs were predicted from the contigs using Prodigal Version 2.6.3^5^ (Chen et al., 2020). Genes ≥100 bp were selected, and the encoded amino acid sequences were predicted.

All gene sequences predicted from the samples were clustered using CD-HIT Version 4.7^6^ (Fu et al., 2012) with 90% identity and coverage as the parameters. The longest gene in each cluster was selected as the representative sequence to construct a non-redundant gene set. SOAPaligner Version soap2.21 release^7^ (Li et al., 2008) was used to align the high-quality reads of each sample with the non-redundant gene set (95% identity), and gene abundance in the corresponding samples was quantified.

Amino acid sequences encoded by the non-redundant gene set were employed to probe the NR database using Diamond Version 2.0.13^8^ (Buchfink et al., 2015), with BLASTP parameters set to e-value ≤1e^−5^. Taxonomic annotation data corresponding to NR were obtained from the taxonomic database, and species abundance was calculated based on the sum of the gene abundances for that species. Diamond Version 2.0.13 was used to align the amino acid sequences to those available in the Kyoto Encyclopedia of Genes and Genomes (KEGG) database (BLASTP, *e*-value ≤ 1e^−5^), and KEGG functions of the corresponding genes were obtained. The abundance of functional categories (KO, Pathway, and Module) was calculated using the sum of gene abundances in each category (Fang et al., 2025).

#### 2.4.2 Metagenic component boxes and taxonomic and functional inferences

The raw data were quality-controlled using fastp Version 0.23.0 (see Footnote 2) to obtain clean, high-quality data. Contigs with lengths ≥1,000 bp after assembly were subjected to sample binning using Metabat Version 2.12.1^9^, CONCOCT Version 0.5.0^10^, and Maxbin Version 2.2.5^11^. Finally, the bins obtained from different tools were merged using DAS_Tool Version 1.1.0^12^ to regenerate the bins, which were then purified using RefineM Version 0.0.24^13^ to obtain the bins redefined as metagenome-assembled genomes (MAGs) (Li et al., 2024). The duplicate MAGs were removed by clustering with the Python program dRep Version 2.2.9^14^. First, the MAGs were partitioned into primary clusters using Mash^15^ with a threshold of First clusters (pa) ≥ 90% Mash ANI. Secondary clustering was performed at a threshold of Secondary clusters (sa) ≥ 99% ANI with a genome overlap of ≥10%. Based on the quality evaluation criteria of CheckM Version 1.0.12^16^, 25 non-redundant MAGs were classified as medium-quality MAGs (completeness ≥50% and contamination <10%) (Liao et al., 2023).

### 2.5 Weighted gene coexpression network analysis (WGCNA)

The weighted coexpression network was constructed for the genes of each internode tissue using the WGCNA package in R. To ensure the stability and accuracy of network construction, genes with zero variance and those with >10% missing samples were filtered. Then, the C–N cycling–related genes across three biological replicates were selected to construct the coexpression matrix. The pickSoftThreshold function of WGCNA was applied to calculate the weighted coefficient “*β*,” evaluating the mean connectivity of genes within the *β* = 2 range. β was selected to achieve a correlation coefficient (*R*^2^) close to 0.8 while maintaining sufficient gene connectivity at the corresponding β value.

The abundances of carbon and nitrogen cycling functional genes were calculated using the fragments per kilobase of exon model per million mapped fragments (FPKM) method (Feng X. et al., 2025; Hu et al., 2022). A gene clustering tree was constructed based on expression correlation, and modules were partitioned from the clustering relationships. Genes with coexpression patterns were assigned to the same module, distinguished by different colors, with a minimum module size of 30 (min Module Size = 30) and a module merging parameter of 0.25 (merge CutHeight = 0.25). Modules were selected based on gene significance (GS) for marked association with GHG emissions, and genes within the associated modules were extracted for differential gene abundance analysis (Li et al., 2024).

### 2.6 Data analysis

Data collection and collation were performed using Excel 2021 (Microsoft Co., WA, United States). SPSS 25 (SPSS Inc., NY, United States) was used to analyze the variations in soil physicochemical properties and microbial biomass C–N under different N fertilizer applications (ANOVA, *p* < 0.05). Sequencing data were analyzed using the Majorbio i-Sanger online cloud platform^17^. The “mothur” V1.30 package in R software was used to calculate the Shannon, Chao1, and Phylogenetic Diversity (Pd) indices. Based on the Bray–Curtis distance, which was used to measure microbial taxonomic differences (β-diversity), we conducted nonmetric multidimensional scaling (NMDS) analysis using the “vegan” package in R software. To determine whether the variations in soil microbial community structure under different N fertilizer application rates were statistically significant (*p* < 0.05), we applied two nonparametric dissimilarity test methods (ANOSIM). The “iCAMP” package in R software was employed to obtain the species specificity-occupancy (SPEC-OCCU) plot and identify potentially key species within the community (Liang et al., 2020). ASVs with specificity and occupancy ≥0.7 in each group (i.e., with group specificity and universality in most samples of the same group) were defined as a specialized species of group (Gweon et al., 2021). The “picante,” “ape,” and “parallel” packages in R software were employed to quantify the deviation between the absolute and random phylogenetic distances of the community using the null model analysis (Ning et al., 2024). The “vegan” package in R software was used for analyzing the neutral community model, indicating the relationship between occurrence frequency and relative abundances of microbial taxa, and establishing the contribution of random processes to the aggregation of microbial communities. The “psych,” “reshape2,” “ggplot2,” “randomForest,” and “patchwork” packages in R software were employed to understand the effects of different soil environmental variables on the abundance of microbial communities and the contribution of these communities to each variable using a Random Forest (RF) model (Jiao et al., 2020). The “vegan,” “dplyr,” “ggplot2,” “ggcor,” and “RColorBrewer” packages in R software were used to reveal the correlations between GHG emissions and C–N functional genes via the Mantel test. The R package (4.4.0) “rfPermute” was used to ascertain the significance of the most vital explanatory variables affecting the response variables via RF model analysis. A structural equation model (partial least squares path model; PLS-PM) was applied to ascertain the feedback mechanisms between microbial diversity and maize C–N footprint under different applications of slow-release N fertilizer. The path coefficients indicated the direction and strength of the linear relationship between latent variables, and PLS-PM was constructed using the “plspm” package in R software (Zhou X. et al., 2024; Zhou Z. et al., 2024).

## 3 Results

### 3.1 Effects of varying N levels on GHG emissions and physicochemical properties

**Figure 2:**
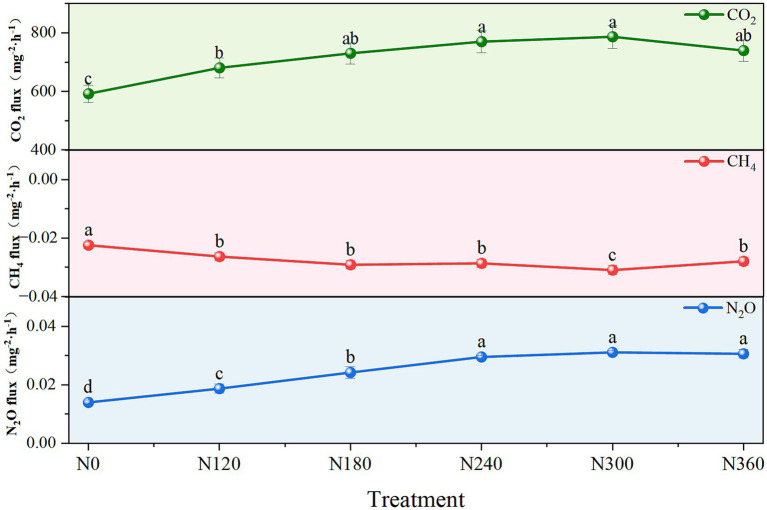
Effects of nitrogen application rate on CO₂, CH₄, and N₂O average emission fluxes in a maize field. The various lowercase letters indicate statistically significant differences among the N application levels at *p* < 0.05.

Application of different N fertilizer levels significantly influenced soil physicochemistry and GHG emissions. The CO₂ and N₂O emissions exhibited a nonlinear response to the addition of N fertilizers, with fluxes increasing up to a certain level before declining. The CH₄ uptake revealed an identical response pattern, thereby indicating an optimal N level for these gaseous fluxes (Figure 2). Furthermore, N application influenced the soil temperature, although the effect of different N levels on soil temperature was insignificant (Supplementary Table S1). With an increase in N fertilizer application rates, the contents of SOC, NO_3_^−^-N, microbial biomass carbon (MBC), and microbial biomass nitrogen (MBN) first increased and then decreased, generally reaching their lowest values under N0 treatment and highest under N240. Soil TN, NO_3_^−^-N, and NH_4_^+^-N contents were directly proportional to the N application rate, with the lowest values under N0 and the maximum values under N360. The soil pH gradually declined with an increase in the N fertilizer application rate, indicating that N fertilizers reduced soil alkalinity (Supplementary Table S1).

### 3.2 GHG emissions and microbial diversity analysis

**Figure 3:**
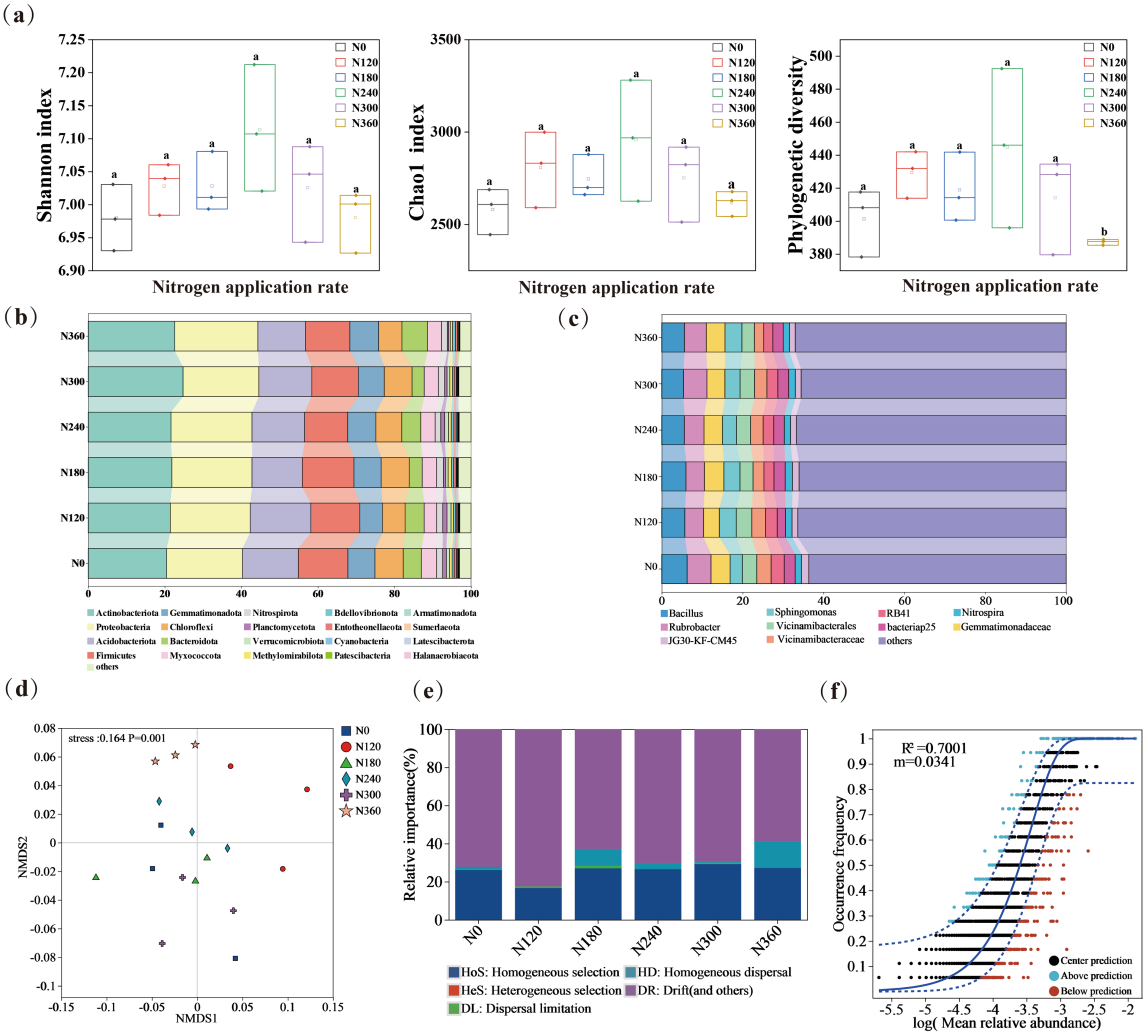
The effects of different nitrogen application levels on the diversity of soil microorganisms. The Shannon index, Chao1 index, Pd (Phylogenetic Diversity) index **(a)**, phylum level dominant bacteria phylum **(b)**, Genus level dominant bacteria genus **(c)** and Non-metric Multidimensional Scaling **(d)** of bacteria under different nitrogen application levels were analyzed, and the community composition process was analyzed through the zero model **(e)** and the neutral model **(f)**.

The community composition at the phylum level fitted using the amplicon and metagenomic sequences revealed high consistency (*R*^2^ = 0.93) (Supplementary Figure S1). The application of N fertilizers remarkably increased bacterial *α*-diversity (*p* < 0.05) (Figure 3a). The bacterial Shannon index (diversity) and Chao1 richness estimator were the lowest under the N0 treatment, but reached their maximum values under the N240 treatment (Figure 3a). Bacterial diversity markedly correlated with N₂O and NH₄^+^-N (Supplementary Figure S2). Compared with N0, the relative abundances of Actinobacteria, Proteobacteria, and Nitrospirota were enhanced, whereas those of phyla such as Methylomirabilota, Acidobacteriota, Chloroflexi, Myxococcota, and Planctomycota declined (Figure 3b and Supplementary Table S2). Actinobacteria members were conspicuously enriched under N application compared with none (Figure 3b and Supplementary Table S2), while the relative abundances of Chloroflexi and Fibrobacterota were markedly reduced (Figure 3b and Supplementary Table S2). In addition, N fertilizer application altered the soil microbial composition, selecting for a dominant bacterial community comprising *Bacillus*, *Rubrobacter*, *Sphingomonas*, *Nitrospira*, and *RB41*—genera that are integral to C and N cycling. Of these, the nitrifier *Nitrospira* was consistently and significantly enriched under N fertilizer addition compared with the N0 treatment, thereby indicating an enhanced nitrification capacity (Figure 3c). The NMDS analysis revealed a clear separation of bacterial communities, indicating that bacterial β-diversity varied significantly across different nitrogen application levels (Figure 3d). Null model analysis revealed that assembly processes of microbial communities under different N fertilizer application rates were primarily governed by stochastic processes (Figure 3e), and the neutral model had a high goodness of fit (*R*^2^ = 0.70) (Figure 3f).

**Figure 4:**
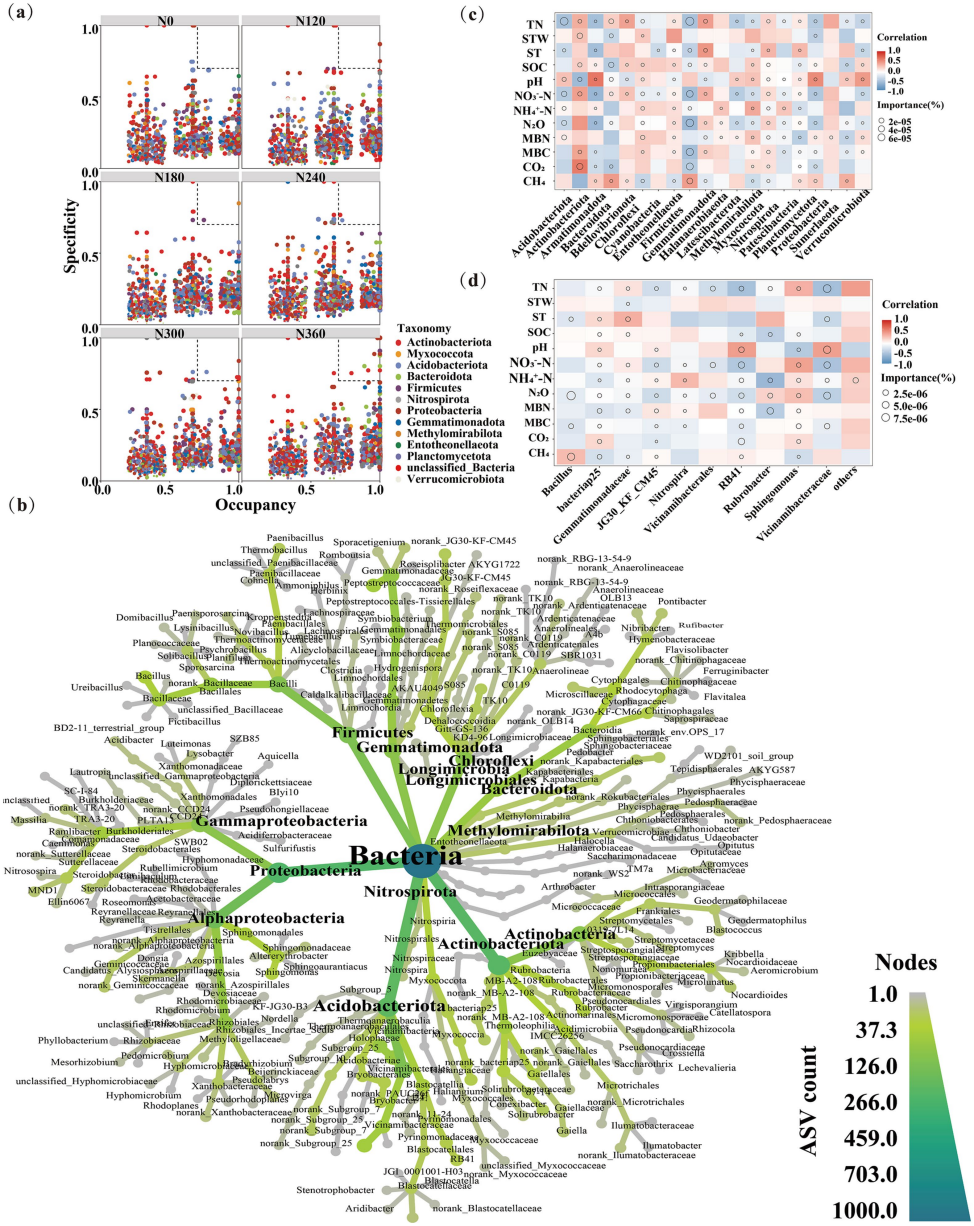
Distribution characteristics of dominant and specialized species under varying nitrogen application levels. The specification-occupation plot illustrates the distribution and specificity of amplicon sequence variants (ASVs) with an average relative abundance exceeding 0.01% in each soil layer. ASVs exhibiting specificity and occupation values ≥0.7 are classified as specialized species **(a)**. The classification tree depicts the quantity and composition of specialized species within bacterial communities, where darker classification units signify a relatively higher number of ASVs within that unit for the respective soil layer **(b)**. The heatmap visualizes the correlations between microbial species at the phylum **(c)** and genus levels **(d)** of microorganism’s abundance and soil environmental variables. In the heatmap, the specific soil environmental variables on the *X*-axis serve as dependent variables in the random forest model, and the abundance of each microbial species is treated as a feature variable. The size of the small circles in the heatmap reflects the importance of each feature variable in the random forest model. ST, soil temperature; SWC, soil water content; SOC, organic carbon; TN, total nitrogen; NH_4_^+^-N, nitrate nitrogen; NO_3_^−^-N stands for ammonium nitrogen; MBC, microbial biomass carbon; and MBN, microbial biomass nitrogen.

The SPEC-OCCU graph showed that ASV occupancy varied remarkably under different N treatments (Figure 4a). The number of specialized species under N0, N120, N180, N240, N300, and N360 treatments was 0, 2, 1, 0, 3, and 6, respectively. They were mainly Proteobacteria and Actinobacteria (Supplementary Table S3), and Bacteroidetes, Proteobacteria, and Actinomycetes with specificity and universality (Figure 4b). Simultaneously, the bacterial taxa and GHG emissions were correlated (Figure 4c). The relative abundances of Methylomirabilota and Nitrospirota were significantly correlated with CH_4_, N_2_O, and CO_2_ emissions (*p* < 0.05). Acidobacteriota and Firmicutes can inhibit N_2_O emissions. Actinobacteria spp. were remarkably positively associated with CO_2_ emissions but negatively with Firmicutes. However, Firmicutes were markedly directly correlated with CH_4_ emissions (Figure 4c). Our results reveal the direct and indirect pathways through which N fertilizers influence soil processes, such as the direct stimulation of nitrification (*Nitrospira*) and N₂O emissions via increased N availability (TN, NO₃^−^-N), which is accompanied by soil acidification, and the indirect pathway where SOC modulates these processes by controlling the microbial biomass levels (MBC and MBN) (Figure 4d).

### 3.3 Functional genes related to the C and N cycles driving GHG emissions

**Figure 5:**
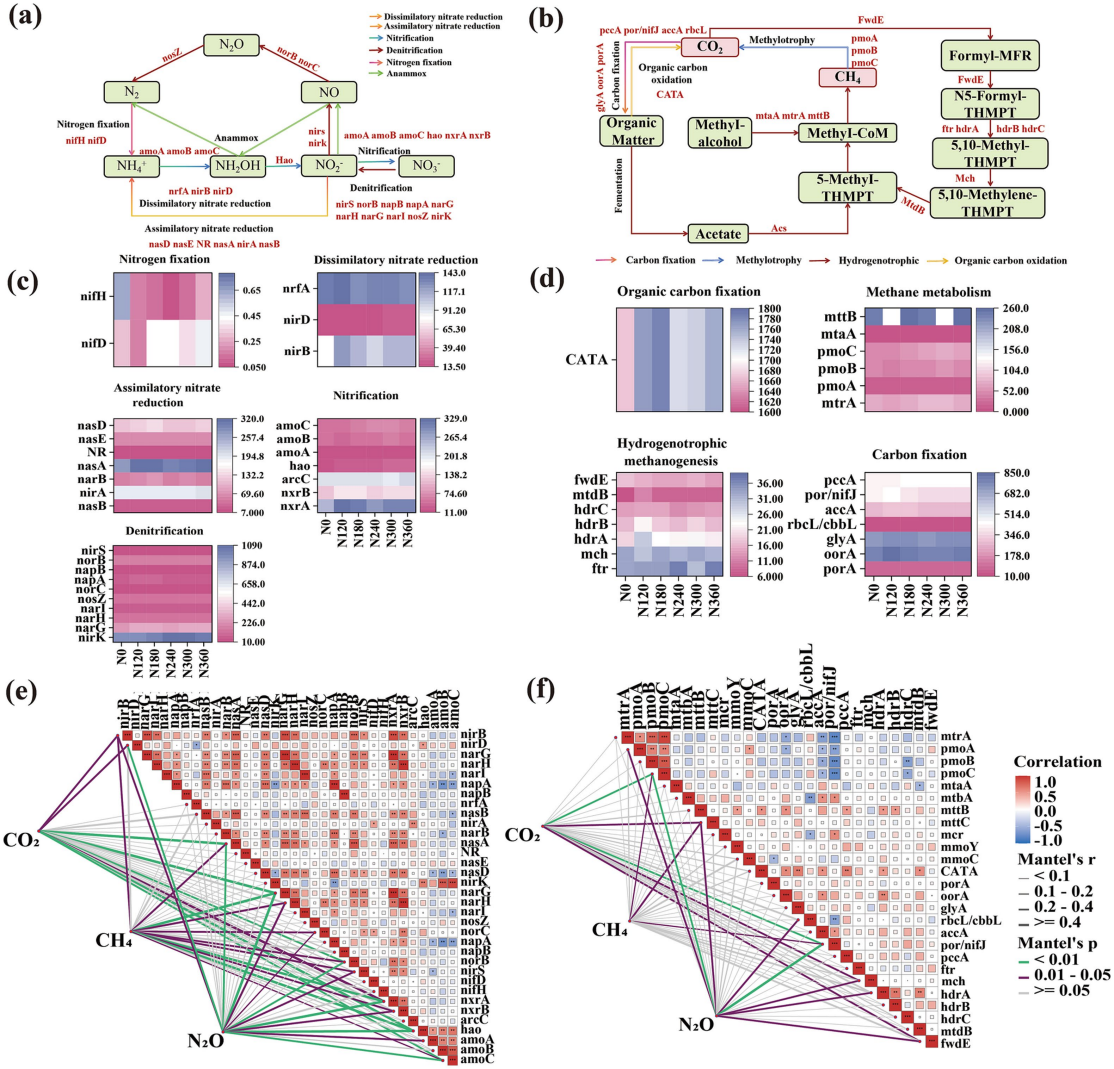
Correlation analysis between carbon-nitrogen cycling functional genes and greenhouse gas emissions in soil. Nitrogen metabolism, carbon metabolism-related processes, and associated functional genes in soil **(a,b)**; correlation analysis between carbon-nitrogen cycling functional genes and GHGs **(c,d)**. Purple and green blocks indicate positive and negative correlations with carbon-nitrogen functional genes, respectively; darker colors represent larger *R* values and stronger correlations. Gray lines represent the relationships between GHGs and functional genes, with thicker and darker lines indicating stronger correlations (**p < 0.01, *p < 0.05) **(e,f)**.

The abundance of C and N cycle associated functional genes was consistent with the pattern of GHG emissions (Figures 5a,b). Compared with N0, the application of N fertilizer enhanced the abundance of *amoA*, *amoB*, *amoC*, and *hao* during nitrification, and the overall performance first increased and then decreased; *amoC* was remarkably correlated with CH_4_ uptake and CO_2_ and N_2_O emissions (Figure 5c). *nirS*, *narG*, and *norB* involved in denitrification were markedly enriched under N fertilizer application, which were significantly associated with CH_4_ uptake and N_2_O emission. *nirB* gene abundance involved in the heterogeneous reduction of nitrate to ammonium was also conspicuously enriched under N fertilizer application. Compared with the control, the gene abundance of *nirB* increased by 51.55, 38.28, 18.23, 35.50, and 35.20% under N120, N180, N240, N300, and N360 treatments, respectively, and was significantly associated with CH_4_ uptake and CO_2_ and N_2_O emissions. In contrast, *nifH* was remarkably downregulated under N fertilizer treatment. Reduced N fixation–related *nifH* and enhanced abundance of denitrification-associated *nxrA* and *nxrAB* may be key factors elevating N_2_O emission (Figure 5b). In addition, C fixation–related functional genes, *por* and *nifj*, were significantly suppressed and associated with CH_4_ uptake and N_2_O and CO_2_ emissions. The abundance of genes involved in hydrogenotrophic methanogenesis, *mch* and *frdE*, was lower under N fertilizer application than under N0 treatment (Figure 4d) and was markedly correlated with CO_2_ and N_2_O emissions. Methane metabolism–associated functional genes, *pmoC* and *mttC*, were conspicuously enriched under N fertilizer application and significantly associated with CH_4_ uptake (Figure 5d).

In the N metabolism pathway, DNRA (*nirB* and *nirD*), ANRA (*nasA*), denitrification (*narG* and *norB*), and nitrification (*nxrA*, *hao*, and *amoC*) associated genes were significantly correlated with CO₂ emissions (*p* < 0.01 and *p* < 0.05). Notably, all these genes were specifically and significantly associated with CH₄ and N₂O emissions (*p* < 0.01 and *p* < 0.05) (Figure 5e). Within the C metabolic framework, methane metabolism (*pmoC* and *mttC*), carbon fixation (*por*/*nifJ*), and hydrogenotrophic methanogenesis (*mch* and *frdE*) genes were remarkably linked with CO₂ emissions (*p* < 0.01 and *p* < 0.05). In particular, *pmoC*, *mttC*, and *por*/*nifJ* were markedly associated with CH₄ emissions (*p* < 0.01 and *p* < 0.05). N₂O emissions were significantly correlated with methane metabolism (*pmoA*, *pmoC*, and *mttC*), carbon fixation (*por*/*nifJ* and *rbcL*/*cbbL*), and hydrogenotrophic methanogenesis (*mch*, *hdrA*, and *frdE*) associated genes abundance (*p* < 0.01 and *p* < 0.05) (Figure 5f).

The WGCNA was used to identify the gene abundance profiles of genes associated with GHG emissions. The genes were clustered based on gene abundance, yielding a network with six modules (Supplementary Figure S3a and Figure 4b). Spearman correlation analysis revealed three significant associations between metagenomic modules and soil physicochemical properties/GHG emissions. The methane emissions were markedly correlated with the key modules MEblue, MEyellow, and MEgrey (*r* = 0.588, *r* = 0.61, and *r* = −0.574; *p* < 0.05); nitrous oxide emissions were remarkably associated with MEblue, MEyellow, and MEgrey (*r* = −0.865, *r* = −0.529, and *r* = 0.513; *p* < 0.05); and methane emissions were significantly correlated with MEblue and MEyellow (*r* = −0.693 and *r* = −0.609; *p* < 0.05) (Supplementary Figure S3c). MM-GS analysis further validated the marked correlations between GHG emissions and modules (Supplementary Figure S4). KEGG database analysis of the blue, orange, and yellow module genes identified 10 key genes driving N cycling (*nrfA*, *napA*, *nrfH*, *nrtC*, *nasD*, *norC*, *hao*, *napB*, *arcC*, *NR*, and *nifH*) (Supplementary Figure S3b and Supplementary Table S4) and 14 vital genes driving C cycling (*por*/*nifJ*, *accA*, *frdB*, *mch*, *hdrC*, *mtbA*, *mcr*, *glyA*, *rbcL*/*cbbL*, *mttC*, *mmoC*, *porA*, *mch*, and *fwdE*) (Supplementary Table S4).

### 3.4 Metagenomic component boxes revealed the core taxonomic groups affecting GHG emissions

**Figure 6:**
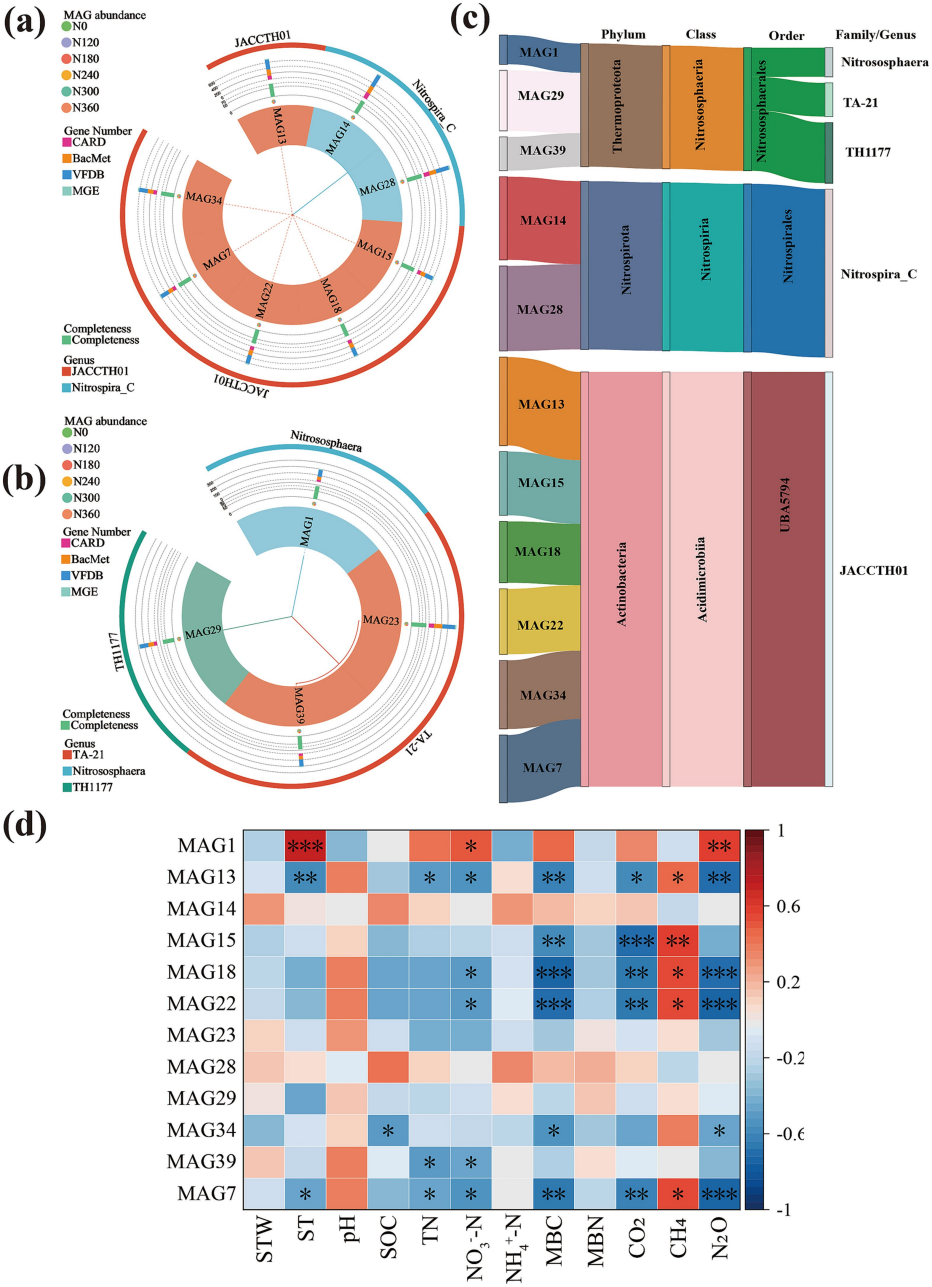
Physicochemical-related heatmaps, phylogenetic and taxonomic characterization of macrogenomic assembled genomes (MAGs). Phylogenetic tree of bacterial **(a)** and archaeal **(b)** MAGs and their completeness. Heatmap of MAGs in relation to physicochemical correlation **(a)**, significant correlation of core MAGs with the taxonomic Sankey map **(c)**; Heatmap of MAGs in relation to physicochemical correlation **(d)**.

In total, 11 MAGs (completeness > 70% and contamination<5%) were obtained (Figure 6 and Supplementary Table S5) after partitioning them into primary and secondary clusters at ≥90% and ≥99% Mash ANI thresholds with genome overlap of ≥10%. These MAGs comprised the dominant phyla Actinobacteria (MAG13, MAG15, MAG18, MAG22, MAG34, and MAG7), Thermoproteota (MAG1, MAG29, and MAG39), and Nitrospirota (MAG14 and MAG28) (Figures 6a–c and Supplementary Table S6). Among them, seven core MAGs (MAG1, MAG13, MAG15, MAG18, MAG22, MAG34, and MAG7) were significantly associated with N₂O, CO₂, and CH₄ emissions (Figure 6d and Supplementary Table S6). KEGG functional annotation indicated that these MAGs belonged to Actinobacteria and Thermoproteota. MAGs such as *narG*, *narH*, *narB*, *narI*, *NapA*, *norB*, *nirK*, *amoB*, and *amoC* (Supplementary Table S7) were related to nitrification, denitrification, and N fixation. In contrast, *mcr*, *acsA*, *pccA*, *ftrA*, *pmoB*, and *pmoC* were associated with CH₄ metabolism and hydrogenotrophic methanogenesis.

### 3.5 Interactive effects of environmental factors and C/N-cycle genes on GHG emissions

**Figure 7:**
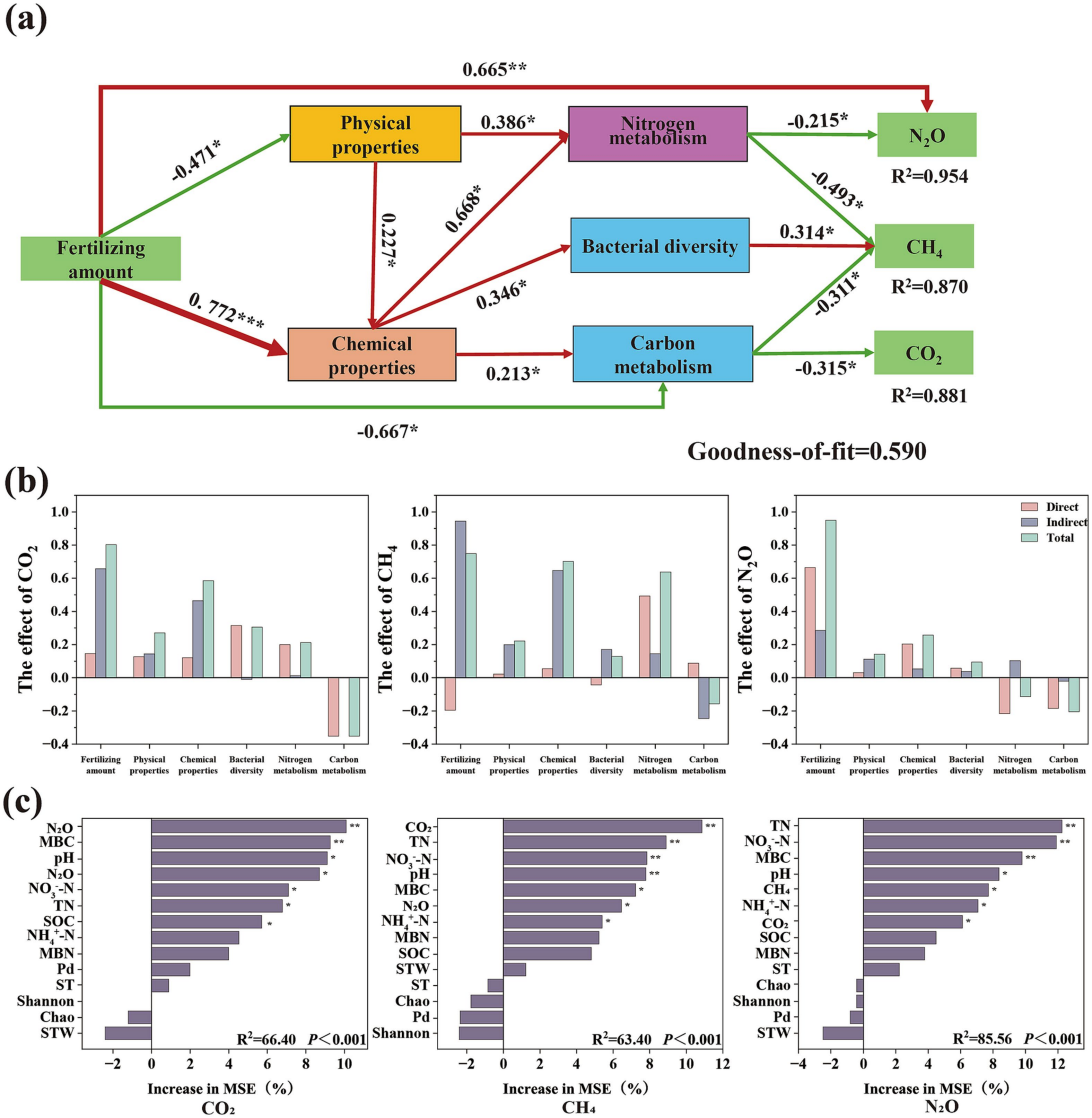
Carbon and nitrogen cycle metabolic genes and soil environmental determinants drive greenhouse gas emissions (goodness of fit = 0.590). PLS-PM was used to resolve the effects of soil environmental factors and microbial interactions on GHG emissions, with thick and thin lines corresponding to standardized regression coefficients, green for negative correlation, red for positive correlation, *p < 0.05, **p < 0.01 **(a)**. Fertilizing amount, physical properties, chemical properties, bacterial diversity, nitrogen metabolism, carbon metabolism direct effect, indirect effect, total effect on GHG emissions **(b)**. Random forests explain the effects of environmental factors on CO_2_, CH_4_, and N_2_O emissions **(c)**.

Partial least squares structural equation modeling (PLS-SEM) was used to analyze how N fertilizer coordinates C–N cycling to drive GHG emissions, thereby revealing the potential pathway by which soil physicochemical properties, microbial diversity, and C/N metabolic processes influence GHG emissions through direct and indirect pathways. N fertilizer application rate not only directly and significantly influenced N₂O emissions but also regulated GHG (CO₂, CH₄, and N₂O) emissions by modulating the soil physicochemical properties, microbial diversity, and C/N metabolic processes (Figure 7a). Analysis of direct, indirect, and total impacts on GHG emissions revealed that N fertilizer application rate had the greatest impact, followed by soil chemical factors, with C and N metabolism also being major contributors (Figure 7b). RF analysis of environmental factors regulating CO₂, CH₄, and N₂O emissions identified total N, pH, NO_3_^−^-N, and microbial biomass C as common driving forces (Figure 7c).

## 4 Discussion

### 4.1 N fertilizer regulates soil nutrients and microbial diversity

Different levels of N fertilizer application affect the soil microenvironment. N fertilizer application increases soil nutrient levels, reduces pH, but does not affect its physical properties (Zhang et al., 2019). In this study, N fertilizers increased soil N content, SOC, and microbial biomass–associated C and N contents. The enhancement in soil C and N levels provided more substrates for soil microbe–associated N cycling, thereby enhancing microbial activity and increasing microbial biomass C and N (Cao et al., 2006). N fertilizer application has been reported to elevate soil TN, NO_3_^−^-N, and NH_4_^+^-N contents (Van Zwieten et al., 2010), which is consistent with the results of this study. Comprehensive analysis indicated that the soil nutrient levels under N240 were reasonable, with the maximal microbial richness, species diversity, and phylogenetic diversity.

Appropriate N fertilizer use can improve the soil physicochemical properties and microbial activity, whereas excessive N fertilizer application reduces soil microbial diversity (Zeng and He, 2016; Sushko et al., 2019). The present study also found that soil nutrient levels were directly proportional to the N fertilizer application rates, and soil microbial diversity (Shannon, Chao1, and Pd indices) first increased and then decreased, indicating that excessive N fertilizer application suppresses soil richness and phylogenetic diversity. The changes in microbial community abundance observed in this study were significantly correlated with changes in soil pH, TN, and NO_3_^−^-N contents. N fertilizers markedly increased soil TN and NO_3_^−^-N contents. Consistent with previous findings (Huang et al., 2023), the relative abundances of Actinobacteriota, Proteobacteria, Nitrospirota, and Firmicutes increased with elevated N fertilizer application rates in our study, whereas those of Acidobacteriota, Chloroflexi, Myxococcota, and Planctomycota were significantly suppressed (Yang et al., 2024). Many of these phyla are integral to soil C and N cycling. Notably, the marked reduction in the abundance of Methylomirabilota a phylum closely associated with methanogenesis under N fertilization, likely constitutes a key microbial mechanism driving the observed enhancement in soil CH₄ uptake.

### 4.2 Interactions between N fertilizer levels and microbial community regulate GHG emissions

Nitrogen fertilization is a key driver of N₂O and CH₄ emissions in agricultural soils. As a direct substrate for N₂O formation, exogenous nitrogen input promotes N₂O production by participating in soil nitrification and denitrification processes (Zhang et al., 2022). Studies have shown that increased nitrogen application not only significantly stimulates N₂O emissions but also weakens the soil CH₄ sink and leads to a rise in CO₂ emissions (Xiao et al., 2024). Furthermore, the accumulation of soil organic carbon (SOC) can further intensify the emission strength of N₂O and CH₄, becoming a core factor influencing greenhouse gas fluxes and crop yield (Guo et al., 2024). The results of this study revealed that GHG emissions were generally directly proportional to N fertilizer application rates, which exerted the maximal influence on GHG emission levels. The observed positive correlation between the N fertilizer application rates and GHG emissions in our study aligns with the established nonlinear relationship between N input and microbial diversity. Moderate N supplementation has been demonstrated to sustain a higher community diversity by alleviating the microbial C limitation, thereby supporting essential ecosystem functions such as organic matter decomposition and nutrient cycling (Zhou et al., 2020). In contrast, excessive N fertilization induces soil acidification and community homogenization, which ultimately reduces biodiversity and restructures the C and N cycling pathways (Hu et al., 2024). This diversity erosion compromises the functional redundancy and stability of microbial communities, thereby potentially amplifying their sensitivity to N-induced GHG emissions (Yang et al., 2023).

The GHG emissions were also remarkably influenced by the microbial diversity. N fertilizers increased the abundance of Actinobacteria, which possibly enhanced CO₂ release via C mineralization, promoted N₂O production through nitrification and denitrification, and accelerated C–N cycling to further elevate GHG emissions (Liang et al., 2022). Proteobacteria and Cyanobacteria are the dominant N-fixing microbial phyla (Wang et al., 2023). Nitrospirota-mediated nitrification is associated with the production of N₂O (Zhang et al., 2023a,b,c; Zhang X. et al., 2023). Planctomycota possess the anammox function, oxidizing NH_4_^+^ with NO_2_^−^ to generate N_2_ gas (Strous et al., 1999). This study found that N fertilizer application increased the abundance of Proteobacteria, Cyanobacteria, and Nitrospirota; decreased the abundance of Planctomycota; and possibly enhanced N₂O emissions. Acidobacteriota, involved in soil organic matter decomposition and nutrient cycling, possibly elevate CO₂ emissions. Low N levels promote CH_4_ emissions, which gradually decline with increasing N fertilizer application (He et al., 2020; Nan et al., 2020; Liao et al., 2021). This may be attributed to the significant reduction in the abundance of Methylomirabilota within the phylum NC10 under nitrogen application. This genus is primarily involved in CH₄ cycling in anaerobic or microoxic environments, potentially through the process of nitrite-dependent anaerobic methane oxidation (Ferry, 2010). This study found that the abundance of Methylomirabilota was significantly correlated with CH_4_ uptake; its abundance decreased after N fertilizer application, thereby increasing CH_4_ uptake.

### 4.3 N fertilizer–based coordination of C–N cycle–associated functional genes abundance drives GHG emissions

N fertilization exerts systematic regulatory effects on the gene abundance of key functional genes that are involved in soil microbial C and N cycling by altering the nutrient availability. Our study demonstrated that N fertilizer application significantly downregulated C fixation–related genes (*por*/*nifJ* and *cbbM*) while upregulating carbohydrate decomposition genes (*amyA*) (Wang et al., 2018). This shift indicates a microbial metabolic strategy transition from energy-consuming C fixation toward more economical decomposition pathways under N input conditions (Ouyang et al., 2018; Enebe and Babalola, 2021). Regarding CH_4_ metabolism, the observed decrease in hydrogenotrophic methanogenesis gene (*mch* and *frdE*) abundance may be attributed to intensified substrate competition from heterotrophic microorganisms for H₂ and CO₂. Conversely, CH_4_ oxidation genes (*pmoC*, *mttC*, and *pmoA*) (Cai and Wang, 2024) were significantly enriched and primarily associated with the relief of N limitation in methanotrophs and optimization of their microniches. The growth of methanotrophs is typically N-limited; thus, N fertilizer application provides readily available ammonium or nitrate, directly enhancing their activity and *pmoC* abundance (Dong and Cai, 2017). Furthermore, N fertilization promotes root-derived exudates such as methanol, which may further stimulate methanotrophic activity (Gao et al., 2016; Chen et al., 2010).

In the N cycle, the increased availability of N via fertilizer application led to the suppression of N fixation genes (*nifH* and *nifA*) as microorganisms preferentially use soil-available N rather than investing energy in the process of biological N fixation (Carey et al., 2016; Guo et al., 2021). Concurrently, nitrification and denitrification processes were strongly activated, where genes encoding ammonia monooxygenase (*amoA*, *amoB*, and *amoC*) and hydroxylamine oxidase (*hao*) were significantly enriched, thereby promoting the conversion of ammonium to nitrite (Du et al., 2024). In this study, the abundances of denitrification-related genes (*nosZ*, *nirS*, *narG*) exhibited an increasing trend with higher nitrogen application rates. Previous studies have demonstrated that nitrogen fertilization is generally associated with enhanced gene abundance of nitrification and denitrification genes abundance (e.g., *amoA*, *nirS*, *nirK*) (Guo et al., 2021), which may, to some extent, accelerate soil nitrogen transformation processes (Chen et al., 2017). In this study, the relative abundance of the nosZ gene decreased under high nitrogen application, and this change coincided with increased N₂O emission fluxes observed during the same period. This finding implies that the decline in nosZ abundance may have weakened the soil’s capacity for N₂O consumption, thereby indirectly influencing net N₂O emissions. The observed shifts in gene abundances were associated with increases in soil organic carbon, total nitrogen, and microbial biomass carbon and nitrogen contents. Overall, nitrogen application suppressed the abundances of genes related to nitrogen degradation, nitrification, and dissimilatory nitrate reduction pathways, while promoting those associated with denitrification and assimilatory nitrate reduction pathways. However, these pathway-level shifts require further validation (Wang J. et al., 2024; Wang X. et al., 2024).

Metagenomic binning analysis further revealed significant enrichment of microbial phyla, including Actinobacteriota, Nitrospirota, and Thermoproteota, which are closely associated with C and N transformations. Of particular interest, the genus Nitrososphaera within Thermoproteota maintained high ammonia oxidation activity even in the acidified soil environment under N fertilization, suggesting the evolution of specific adaptive metabolic mechanisms to cope with low pH stress (Wang et al., 2014). Integrated analysis revealed that soil pH, TN, microbial biomass C, and NO_3_^−^-N content are not only key environmental drivers of GHG emissions but are also significantly correlated with key co-occurrence network modules (blue, orange, and yellow modules). This implies that environmental factors interact synergistically with microbial functional modules to regulate the gene abundance patterns of C- and N-cycling genes and ultimately influence GHG fluxes. These findings reveal a multilevel regulatory network that connects “environmental factors–microbial modules–functional genes–gas emissions,” providing new insights into the microbial mechanisms through which N fertilizers regulate GHG emissions from agricultural soils.

## 5 Conclusion

In conclusion, nitrogen application rates significantly altered soil physicochemical properties, leading to a decrease in soil pH and a marked increase in the contents of soil organic carbon, total nitrogen, ammonium nitrogen, and nitrate nitrogen, particularly under the N240 treatment. These environmental changes drove shifts in the soil microbial community structure. This was manifested by an increase in microbial *α*-diversity and a significant enrichment of key microbial phyla with carbon and nitrogen metabolic potential, including Actinobacteriota, Thermoproteota, and Nitrospirota. Metagenomic analysis further revealed that the succession of these microbial communities was closely linked to changes in the abundance of key functional genes involved in nitrification (*amoA*, *amoB*, *amoC*, *hao*), denitrification (*nirS*, *narG*, *norB*), and methane metabolism (*pmoC*, *mttC*). Notably, a strong correlation was observed between the abundance of denitrification genes and N₂O emission fluxes, suggesting that denitrification may be a primary pathway contributing to N₂O production in this agro-pastoral ecotone. Furthermore, Actinobacteria, Thermoproteota, and Nitrospirota were identified as the core microbial taxa harboring these functional genes and potentially mediating greenhouse gas emissions. This study elucidates the dynamic patterns of soil carbon and nitrogen cycling metabolic potential in the agroecosystem of the ecotone under varying nitrogen levels and links the abundance of key functional genes to greenhouse gas emission characteristics. These findings provide a scientific basis for optimizing nitrogen fertilization to regulate microbially driven greenhouse gas emissions. Notably, significant changes in the abundance of relevant functional genes and greenhouse gas emission potential were observed at the N240 application rate, suggesting that this nitrogen level may represent a critical threshold for regulating microbial processes. Therefore, in future regional nitrogen management, it is recommended to integrate crop requirements with microbial response characteristics to optimize nitrogen inputs, thereby sustaining productivity while mitigating greenhouse gas emissions.

## Data Availability

The datasets presented in this study can be found in online repositories. The names of the repository/repositories and accession number(s) can be found below: The raw sequences were deposited in the SRA database (Accession Nos. PRJNA1291659 and PRJNA1293101).

## Footnotes

1
https://www.drive5.com/usearch/

2
https://github.com/OpenGene/fastp

3
http://bio-bwa.sourceforge.net

4
https://github.com/voutcn/megahit

5
https://github.com/hyattpd/Prodigal

6
http://weizhongli-lab.org/cd-hit/

7
https://github.com/ShujiaHuang/SOAPaligner

8
https://github.com/bbuchfink/diamond

9
https://bitbucket.org/berkeleylab/metabat

10
https://github.com/BinPro/CONCOCT

11
https://sourceforge.net/projects/maxbin/

12
https://github.com/cmks/DAS_Tool

13
https://github.com/wwood/RefineM

14
https://github.com/BenLangmead/bowtie2/releases/tag/v2.2.9

15
https://mash.readthedocs.io/en/latest/#

16
https://github.com/Ecogenomics/CheckM

17
https://www.i-sanger.com/
