# Supplementary material for: Mechanistic insights into nitrogen fertilizer regulation of carbon-nitrogen cycling and greenhouse gas emissions: a metagenomics-based investigation
Source: Front Microbiol. 2026 Apr 17;17:1808047. doi: 10.3389/fmicb.2026.1808047 (PMC13132864; doi:10.3389/fmicb.2026.1808047)
Supplement: Supplementary file 1 [file Table_1.DOCX]

Supplementary Material

Mechanistic Insights into Nitrogen Fertilizer Regulation of Carbon-Nitrogen Cycling and Greenhouse Gas Emissions: A Metagenomics-Based Investigation

Tiantian Meng^1,2^, Jingjing Shi^3^, Xiangqian Zhang^2,4,5*^, Xiaoyu Zhao^2^, Yanan Liu^1,2^, Meiren Rong^2^, Liyu Chen^2^, Yu Dai^3^, Shuli Wei^2^, Jiawei Liu^2^, Zhanyuan Lu^1,2,3,4,5*^

^1^College of Agronomy, Hebei Agricultural University, Baoding, 071000, China

^2^Inner Mongolia Academy of Agricultural and Animal Husbandry Sciences, Hohhot, 010031, China

^3^School of Life Science, Inner Mongolia University, Hohhot, 010031, China

^4^Inner Mongolia Key Laboratory of Degradation Farmland Ecological Restoration and Pollution Control, Hohhot, 010031, China

^5^Key Laboratory of Black Soil Protection and Utilization (Hohhot), Ministry of Agriculture and Rural Affairs, Hohhot, 010031, China

*** Correspondence:**Zhanyuan Lu

[lzhy2811@163.com](mailto:lzhy2811@163.commail@uni.edu)

Xiangqian Zhang

[zhangxiangqian_2008@126.com](mailto:zhangxiangqian_2008@126.com)

## Supplementary Figures


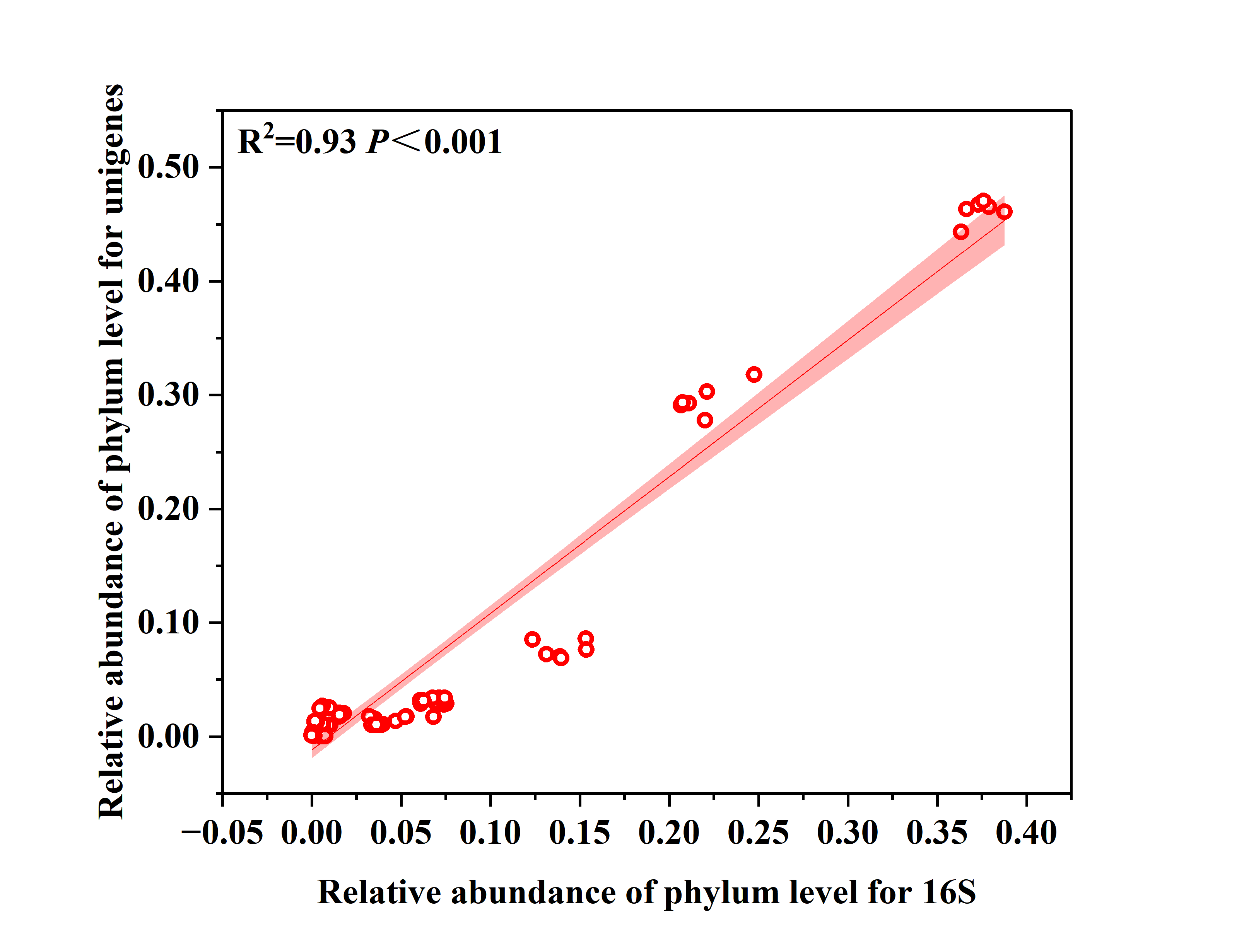


**Supplementary Figure** 1 Spearman’s correlation with relative abundance of phylum level for 16S and Meta.


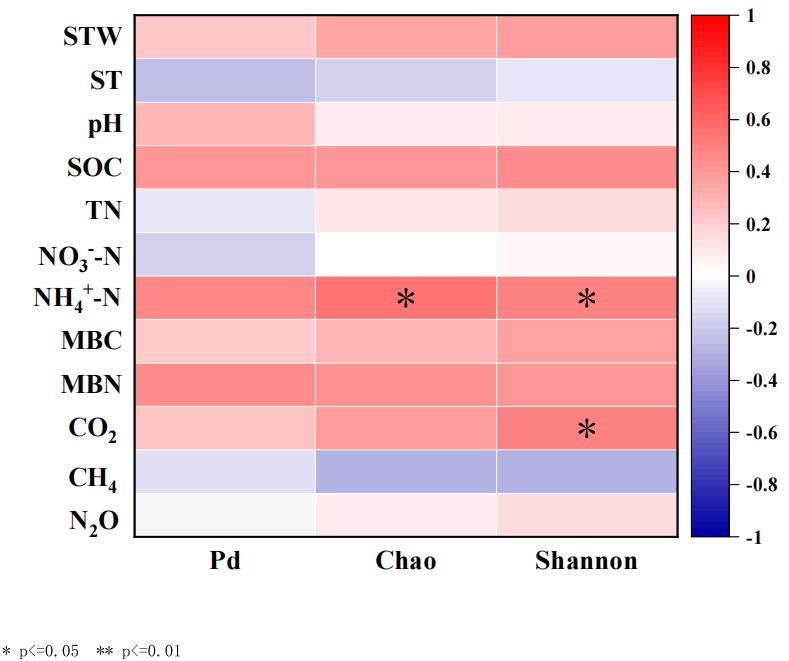


**Supplementary Figure** 2. Correlation analysis between microbial diversity index and environmental factors. * represents *P*<0.05. relationship between the module and greenhouse gas emissions, (*P*<0.05 represents a significant correlation between species/function).


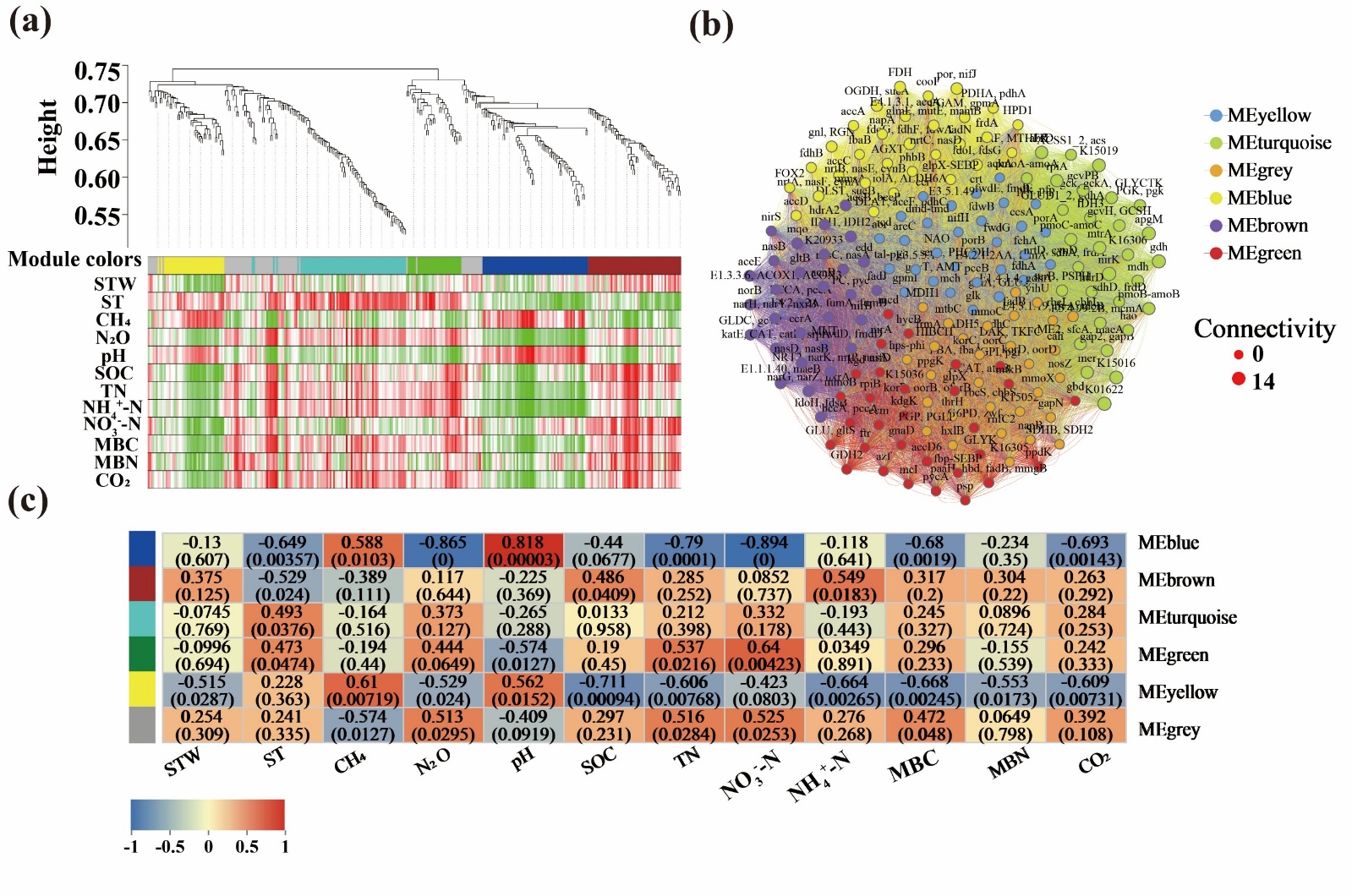


**Supplementary Figure** 3 Weighted gene co-expression network analysis. Clustering dendrogram, this figure can be viewed in two parts: the top half is a hierarchical clustering dendrogram of genes, and the bottom half is gene modules, or network modules. Each colour represents a module, grey represents the inside genes do not belong to any module (a); weighted gene co-expression network (b); physicochemical factors and highly correlated gene modules (c), each set of data in the heatmap on the right side represents the correlation coefficients of the modules with the phenotypes and the significance P-value (in parentheses) (Spearman's correlation), and the modules with the highest correlations and the more significant P-values are filtered to be used as the phenotypes of the Characteristic Modules.


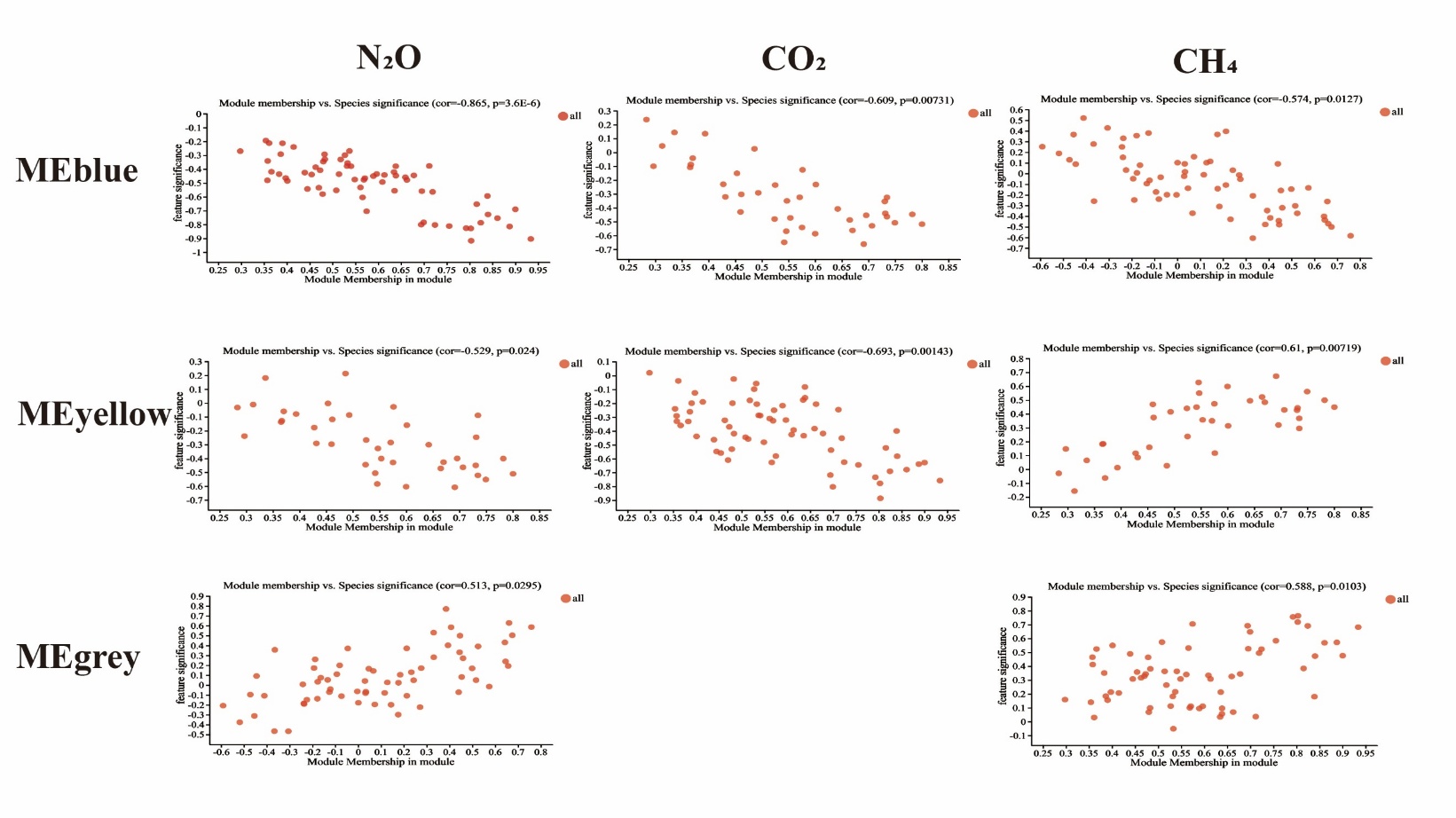


**Supplementary Figure** 4. MM-GS analysis of key modules and advantage modules

Note: The correlation coefficient between each species/function in the module and a phenotype data in the module was calculated to obtain the species/function significance value, and the MM-GS scatter plot was obtained by combining the MM value (i.e., kME) of each species/function in the module, the stronger the correlation between the two, the closer the

## Supplementary Table

**Supplementary Table** 1 Effects of different nitrogen application levels on soil characteristics

| Treatment | N0 | N120 | N180 | N240 | N300 | N301 | F-value |
| --- | --- | --- | --- | --- | --- | --- | --- |
| STW(％) | 11.84±0.47a | 12.27±0.38a | 12.72±0.35a | 12.42±0.5a | 12.41±0.46a | 12.1±0.11a | 1.7 |
| ST (℃) | 20.61±0.1ab | 20.2±0.09c | 20.39±0.19b | 20.58±0.26ab | 20.64±0.13ab | 20.84±0.18a | 5.31** |
| pH | 8.15±0.01a | 8.06±0.04b | 7.95±0.01c | 7.96±0.03c | 7.93±0.04c | 7.84±0.02d | 39.93*** |
| SOC(g·kg^-1^) | 11.84±0.23d | 13.74±0.13c | 14.85±0.08a | 15.07±0.12a | 14.32±0.09b | 13.86±0.38c | 100.14** |
| TN(g·kg^-1^) | 1.05±0.01e | 1.2±0.01d | 1.28±0c | 1.36±0.03b | 1.35±0b | 1.41±0.01a | 254.72*** |
| NO_3_^-^-N(mg·kg^-1^) | 14.16±0.23f | 18.8±0.41e | 27.43±0.14d | 30.3±0.76c | 34.6±1.14b | 44.95±1.44a | 527.08*** |
| NH_4_^+^-N(mg·kg^-1^) | 5.42±0.12d | 5.82±0.15b | 6.2±0.1a | 6.02±0.12a | 5.82±0.13b | 5.71±0.07c | 15.32*** |
| MBC (mg·kg^-1^) | 116.36±4.94d | 181.11±4.37c | 210.54±8.55b | 245.85±8.76a | 241.48±2.52a | 217.64±6.51b | 172.04*** |
| MBN (mg·kg^-1^) | 56.1±0.69d | 63.26±4.29bc | 74.99±6.29a | 70.4±3.05a | 64.57±4.56b | 61.66±3.16c | 8.12** |
| CO_2_(mg·m^-2^·h^-1^) | 591.83±34.85c | 680.46±41.6b | 729.84±8.12ab | 769.98±55.94a | 786.33±4.54a | 739.22±31.72ab | 12.70*** |
| CH_4_(mg·m^-2^·h^-1^) | -0.0224±0.0017a | -0.0263±0.0007b | -0.0291±0.001ab | -0.0286±0.0016b | -0.0309±0.0017c | -0.0279±0.0009b | 14.52*** |
| N_2_O(mg·m^-2^·h^-1^) | 0.014±0.0008d | 0.0187±0.0016c | 0.0242±0.002b | 0.0295±0.0005a | 0.0311±0.0006a | 0.0306±0.0015a | 88.069*** |

**Supplementary Table** 2: Proportion of different phyla abundance under different nitrogen application levels

| **Phylum** | **N0** | **N120** | **N180** | **N240** | **N300** | **N360** |
| --- | --- | --- | --- | --- | --- | --- |
| Actinobacteriota | 0.2047 | 0.2142 | 0.2185 | 0.2161 | 0.2476 | 0.2257 |
| Proteobacteria | 0.1979 | 0.2088 | 0.2093 | 0.2116 | 0.1982 | 0.2175 |
| Acidobacteriota | 0.1462 | 0.1585 | 0.1517 | 0.1377 | 0.1381 | 0.1244 |
| Firmicutes | 0.1295 | 0.1279 | 0.1349 | 0.1125 | 0.1768 | 0.1158 |
| Gemmatimonadota | 0.0706 | 0.0592 | 0.0712 | 0.0736 | 0.0678 | 0.0752 |
| Chloroflexi | 0.0740 | 0.0591 | 0.0732 | 0.0677 | 0.0728 | 0.0608 |
| Bacteroidota | 0.0474 | 0.0499 | 0.0340 | 0.0496 | 0.0316 | 0.0665 |
| Myxococcota | 0.0398 | 0.0327 | 0.0375 | 0.0376 | 0.0369 | 0.0369 |
| Nitrospirota | 0.0158 | 0.0159 | 0.0179 | 0.0147 | 0.0161 | 0.0153 |
| Planctomycetota | 0.0099 | 0.0110 | 0.0062 | 0.0104 | 0.0066 | 0.0035 |
| Verrucomicrobiota | 0.0080 | 0.0107 | 0.0061 | 0.0100 | 0.0073 | 0.0042 |
| Methylomirabilota | 0.0068 | 0.0064 | 0.0085 | 0.0067 | 0.0069 | 0.0057 |
| Bdellovibrionota | 0.0053 | 0.0052 | 0.0058 | 0.0071 | 0.0045 | 0.0075 |
| Entotheonellaeota | 0.0064 | 0.0030 | 0.0066 | 0.0058 | 0.0057 | 0.0057 |
| Cyanobacteria | 0.0018 | 0.0029 | 0.0018 | 0.0027 | 0.0017 | 0.0018 |
| Patescibacteria | 0.0027 | 0.0015 | 0.0018 | 0.0024 | 0.0017 | 0.0023 |
| Armatimonadota | 0.0012 | 0.0013 | 0.0011 | 0.0012 | 0.0006 | 0.0004 |
| Sumerlaeota | 0.0008 | 0.0011 | 0.0003 | 0.0007 | 0.0008 | 0.0009 |
| Latescibacterota | 0.0007 | 0.0007 | 0.0004 | 0.0006 | 0.0007 | 0.0003 |
| Halanaerobiaeota | 0.0004 | 0.0005 | 0.0006 | 0.0005 | 0.0008 | 0.0004 |
| NB1-j | 0.0007 | 0.0005 | 0.0008 | 0.0005 | 0.0003 | 0.0002 |
| Desulfobacterota | 0.0005 | 0.0004 | 0.0006 | 0.0005 | 0.0004 | 0.0005 |
| Fibrobacterota | 0.0010 | 0.0002 | 0.0004 | 0.0004 | 0.0004 | 0.0002 |
| Elusimicrobiota | 0.0004 | 0.0004 | 0.0004 | 0.0006 | 0.0004 | 0.0003 |
| SAR324_cladeMarine_group_B | 0.0004 | 0.0002 | 0.0003 | 0.0004 | 0.0003 | 0.0004 |
| WS2 | 0.0002 | 0.0002 | 0.0002 | 0.0003 | 0.0004 | 0.0002 |
| Deinococcota | 0.0003 | 0.0001 | 0.0001 | 0.0001 | 0.0002 | 0.0003 |
| Abditibacteriota | 0.0001 | 0.0002 | 0.0001 | 0.0002 | 0.0001 | 0.0001 |
| Dependentiae | 0.0001 | 0.0000 | 0.0002 | 0.0001 | 0.0003 | 0.0000 |
| WPS-2 | 0.0001 | 0.0002 | 0.0000 | 0.0001 | 0.0002 | 0.0001 |
| GAL15 | 0.0000 | 0.0001 | 0.0002 | 0.0001 | 0.0001 | 0.0001 |
| RCP2-54 | 0.0001 | 0.0000 | 0.0000 | 0.0001 | 0.0000 | 0.0000 |
| Hydrogenedentes | 0.0001 | 0.0000 | 0.0000 | 0.0000 | 0.0000 | 0.0000 |
| MBNT15 | 0.0000 | 0.0000 | 0.0000 | 0.0001 | 0.0000 | 0.0000 |
| Dadabacteria | 0.0000 | 0.0000 | 0.0000 | 0.0000 | 0.0000 | 0.0000 |
| others | 0.0263 | 0.0267 | 0.0297 | 0.0272 | 0.0287 | 0.0266 |

**Supplementary Table** 3 Specialized species under different nitrogen application treatments

| Group | OTU | Specificity | Occupancy | Abundance_mean | Taxonomy |
| --- | --- | --- | --- | --- | --- |
| N120 | ASV4998 | 0.869048 | 1 | 24.33333 | Proteobacteria |
| N120 | ASV2970 | 0.75 | 1 | 16 | Proteobacteria |
| N180 | ASV469 | 0.848485 | 1 | 18.66667 | Methylomirabilota |
| N300 | ASV5047 | 0.725 | 1 | 19.33333 | Actinobacteriota |
| N300 | ASV15965 | 0.714286 | 1 | 16.66667 | Actinobacteriota |
| N300 | ASV9341 | 0.83871 | 1 | 17.33333 | Proteobacteria |
| N360 | ASV15739 | 1 | 1 | 59.33333 | Proteobacteria |
| N360 | ASV930 | 0.908451 | 1 | 43 | Firmicutes |
| N360 | ASV7262 | 0.877193 | 1 | 33.33333 | Bacteroidota |
| N360 | ASV5573 | 0.802326 | 1 | 23 | Bacteroidota |
| N360 | ASV366 | 0.772727 | 1 | 17 | Actinobacteriota |
| N360 | ASV7323 | 0.887097 | 1 | 18.33333 | Proteobacteria |

**Supplementary Table** 4: Key genes and GS values of greenhouse gas emissions

|  | Module | GS-N_2_O | GS-CH_4_ | GS-CO_2_ |
| --- | --- | --- | --- | --- |
| nrfA | MEblue | -0.329 | 0.381 | -0.419 |
| napA | MEblue | -0.459 | 0.326 | -0.383 |
| nrfH | MEblue | -0.194 | 0.14 | -0.24 |
| nrtC/nasD | MEblue | -0.58 | 0.464 | -0.531 |
| norC | MEgrey | 0.13 | -0.429 | 0.162 |
| HAO | MEgrey | -0.108 | 0.396 | -0.438 |
| napB | MEgrey | -0.0964 | 0.129 | 0.0127 |
| arcC | MEyellow | -0.0866 | 0.415 | -0.292 |
| NR | MEyellow | -0.128 | 0.184 | -0.0875 |
| nifH | MEyellow | -0.401 | 0.357 | -0.473 |
| Por/nifJ | MEblue | -0.904 | 0.682 | -0.758 |
| accA | MEblue | -0.356 | -0.0514 | -0.206 |
| frdB | MEblue | -0.543 | 0.308 | -0.548 |
| mch | MEblue | -0.45 | 0.095 | -0.217 |
| hdrC2 | MEblue | -0.475 | 0.308 | -0.481 |
| mtbA | MEblue | -0.465 | 0.111 | -0.25 |
| mcr | MEblue | -0.464 | 0.213 | -0.125 |
| glyA | MEgrey | 0.165 | -0.138 | 0.249 |
| rbcL, cbbL | MEgrey | 0.769 | -0.478 | 0.642 |
| mttC | MEgrey | 0.104 | -0.309 | 0.394 |
| mmoC | MEgrey | 0.499 | -0.48 | 0.441 |
| porA | MEyellow | -0.16 | 0.314 | -0.232 |
| mch | MEyellow | -0.118 | 0.374 | -0.303 |
| fwdE | MEyellow | -0.445 | 0.44 | -0.481 |

**Supplementary Table** 5 Information of the filtered MAGs

| MAG Id | Domain | GC (%) | Contigs Num | Genome size（bp） | N50 （contigs）（bp） | N90 （contigs）（bp） | Completeness (%) |
| --- | --- | --- | --- | --- | --- | --- | --- |
| MAG42 | Archaea | 29.1 | 469 | 1380157 | 3147 | 1717 | 66.83 |
| MAG39 | Archaea | 35.76 | 314 | 1424005 | 5584 | 2209 | 84.35 |
| MAG23 | Archaea | 36.28 | 723 | 2637620 | 4228 | 1854 | 95.74 |
| MAG26 | Archaea | 44.3 | 308 | 1110118 | 3985 | 2050 | 76.84 |
| MAG20 | Archaea | 36.46 | 481 | 1871180 | 4687 | 1935 | 88.24 |
| MAG17 | Archaea | 44.54 | 295 | 1132180 | 4259 | 2160 | 78.78 |
| MAG21 | Bacteria | 57.18 | 1010 | 2705025 | 3012 | 1515 | 78.67 |
| MAG28 | Bacteria | 57.34 | 797 | 2653800 | 3670 | 1925 | 82.66 |
| MAG29 | Archaea | 28.98 | 566 | 1855379 | 3538 | 1853 | 72.42 |
| MAG16 | Archaea | 43.78 | 348 | 1204402 | 3783 | 2032 | 72.82 |
| MAG12 | Bacteria | 63.18 | 1070 | 2156615 | 2220 | 1161 | 68.95 |
| MAG7 | Bacteria | 63.1 | 633 | 1945653 | 3351 | 1847 | 74.79 |
| MAG34 | Bacteria | 63.14 | 641 | 2021475 | 3579 | 1707 | 72.08 |
| MAG14 | Bacteria | 57.33 | 777 | 2547741 | 3561 | 1921 | 77.12 |
| MAG35 | Archaea | 42.71 | 458 | 1546162 | 3694 | 1937 | 70.74 |
| MAG5 | Bacteria | 63.08 | 707 | 1581489 | 2440 | 1338 | 60.56 |
| MAG1 | Archaea | 43.39 | 272 | 1294978 | 5682 | 2408 | 82.04 |
| MAG9 | Archaea | 43.56 | 367 | 1361070 | 4244 | 2108 | 79.13 |
| MAG3 | Bacteria | 68.52 | 363 | 1252361 | 3610 | 2261 | 57.39 |
| MAG25 | Bacteria | 63.1 | 576 | 1493339 | 2670 | 1664 | 51.29 |
| MAG33 | Bacteria | 63.11 | 537 | 1414386 | 2735 | 1711 | 52.18 |
| MAG37 | Archaea | 44.72 | 241 | 820535 | 3685 | 1981 | 71.88 |
| MAG10 | Archaea | 45.04 | 221 | 829671 | 4330 | 2065 | 71.45 |
| MAG22 | Bacteria | 63.17 | 612 | 1984595 | 3568 | 1863 | 77.75 |
| MAG40 | Archaea | 29.13 | 444 | 1218545 | 2899 | 1692 | 67.24 |
| MAG32 | Bacteria | 63.07 | 717 | 1542699 | 2305 | 1300 | 61.15 |
| MAG38 | Bacteria | 63.11 | 613 | 1847476 | 3220 | 1808 | 66.67 |
| MAG19 | Archaea | 44.97 | 211 | 711170 | 3667 | 2005 | 61.84 |
| MAG8 | Bacteria | 63.12 | 520 | 1345991 | 2668 | 1692 | 58.13 |
| MAG15 | Bacteria | 63.07 | 596 | 1827030 | 3375 | 1816 | 74.12 |
| MAG13 | Bacteria | 63.25 | 826 | 2062202 | 2945 | 1309 | 71.48 |
| MAG44 | Bacteria | 69.97 | 813 | 2242389 | 3017 | 1697 | 63.22 |
| MAG30 | Bacteria | 62.96 | 702 | 1358459 | 2067 | 1204 | 50.59 |
| MAG27 | Bacteria | 57.52 | 986 | 2234373 | 2258 | 1650 | 57.97 |
| MAG2 | Bacteria | 63.03 | 548 | 1421553 | 2698 | 1674 | 52.99 |
| MAG6 | Bacteria | 63.18 | 1048 | 2215829 | 2382 | 1173 | 76.67 |
| MAG4 | Bacteria | 62.97 | 744 | 1551250 | 2306 | 1228 | 61.99 |
| MAG24 | Bacteria | 70.05 | 824 | 2503564 | 3231 | 1837 | 65.81 |
| MAG11 | Archaea | 28.75 | 435 | 1125285 | 2708 | 1668 | 59.76 |
| MAG43 | Archaea | 44.07 | 343 | 1250289 | 3897 | 2151 | 74.76 |
| MAG41 | Archaea | 44.07 | 347 | 1315663 | 4165 | 2137 | 77.61 |
| MAG36 | Archaea | 35.06 | 360 | 1278248 | 4051 | 2083 | 60.19 |
| MAG18 | Bacteria | 63.16 | 574 | 1887524 | 3695 | 1976 | 71.21 |
| MAG45 | Bacteria | 69.98 | 917 | 2762198 | 3248 | 1772 | 70.06 |
| MAG31 | Archaea | 28.9 | 478 | 1393284 | 3096 | 1723 | 65.75 |

**Supplementary Table** 6 Bacterial classification of bins

| MAG Id | Domain | Phylum | Class | Order | Family | Genus |
| --- | --- | --- | --- | --- | --- | --- |
| MAG1 | Archaea | Thermoproteota | Nitrososphaeria | Nitrososphaerales | Nitrososphaeraceae | Nitrososphaera |
| MAG13 | Bacteria | Actinomycetota | Acidimicrobiia | UBA5794 | ZC4RG35 | JACCTH01 |
| MAG14 | Bacteria | Nitrospirota | Nitrospiria | Nitrospirales | Nitrospiraceae | Nitrospira_C |
| MAG15 | Bacteria | Actinomycetota | Acidimicrobiia | UBA5794 | ZC4RG35 | JACCTH01 |
| MAG18 | Bacteria | Actinomycetota | Acidimicrobiia | UBA5794 | ZC4RG35 | JACCTH01 |
| MAG22 | Bacteria | Actinomycetota | Acidimicrobiia | UBA5794 | ZC4RG35 | JACCTH01 |
| MAG28 | Bacteria | Nitrospirota | Nitrospiria | Nitrospirales | Nitrospiraceae | Nitrospira_C |
| MAG29 | Archaea | Thermoproteota | Nitrososphaeria | Nitrososphaerales | Nitrososphaeraceae | TH1177 |
| MAG34 | Bacteria | Actinomycetota | Acidimicrobiia | UBA5794 | ZC4RG35 | JACCTH01 |
| MAG39 | Archaea | Thermoproteota | Nitrososphaeria | Nitrososphaerales | Nitrososphaeraceae | TA-21 |
| MAG7 | Bacteria | Actinomycetota | Acidimicrobiia | UBA5794 | ZC4RG35 | JACCTH01 |

**Supplementary Table** 7 The carbon cycle and nitrogen cycle genes contained in the key MAGs

| Carbon cycle | | | Nitrogen cycle | | |
| --- | --- | --- | --- | --- | --- |
| MAG ID | Gene | Number | MAG ID | Gene | Number |
| MAG18 | mcr | 1 | MAG1 | napA | 1 |
| MAG7 | mcr | 1 | MAG18 | narB | 1 |
| MAG13 | mcr | 2 | MAG7 | narB | 1 |
| MAG15 | mcr | 1 | MAG18 | nasE | 1 |
| MAG18 | acsA | 1 | MAG7 | nasE | 1 |
| MAG7 | acsA | 1 | MAG1 | nasE | 1 |
| MAG13 | acsA | 1 | MAG18 | narB | 1 |
| MAG18 | pccA | 1 | MAG15 | nasE | 1 |
| MAG13 | pccA | 1 | MAG1 | nirK | 3 |
| MAG1 | pmoB | 1 | MAG13 | nirK | 1 |
| MAG1 | pmoC | 1 | MAG15 | nirK | 1 |
| MAG13 | mcr | 2 | MAG13 | norB | 1 |
|  |  |  | MAG1 | amoB | 1 |
|  |  |  | MAG1 | amoC | 1 |
